# NEDDylation promotes stress granule assembly

**DOI:** 10.1038/ncomms12125

**Published:** 2016-07-06

**Authors:** Aravinth Kumar Jayabalan, Anthony Sanchez, Ra Young Park, Sang Pil Yoon, Gum-Yong Kang, Je-Hyun Baek, Paul Anderson, Younghoon Kee, Takbum Ohn

**Affiliations:** 1 Department of Cellular & Molecular Medicine, College of Medicine, Chosun University, Gwangju 61452, Republic of Korea; 2 Department of Cell Biology, Microbiology, and Molecular Biology, College of Arts and Sciences, University of South Florida, Tampa, Florida 33620, USA; 3 Department of Anatomy, School of Medicine, Jeju National University, Jeju-Do 690-756, Republic of Korea; 4 Diatech Korea Co, Ltd, Saemal-ro 5-gil, Songpa-gu, Seoul 05807, Republic of Korea; 5 Division of Rheumatology, Immunology and Allergy, Brigham and Women's Hospital, Smith652, One Jimmy Fund Way, Boston, Massachusetts 02115, USA

## Abstract

Stress granules (SGs) harbour translationally stalled messenger ribonucleoproteins and play important roles in regulating gene expression and cell fate. Here we show that neddylation promotes SG assembly in response to arsenite-induced oxidative stress. Inhibition or depletion of key components of the neddylation machinery concomitantly inhibits stress-induced polysome disassembly and SG assembly. Affinity purification and subsequent mass-spectrometric analysis of Nedd8-conjugated proteins from translationally stalled ribosomal fractions identified ribosomal proteins, translation factors and RNA-binding proteins (RBPs), including SRSF3, a previously known SG regulator. We show that SRSF3 is selectively neddylated at Lys85 in response to arsenite. A non-neddylatable SRSF3 (K85R) mutant do not prevent arsenite-induced polysome disassembly, but fails to support the SG assembly, suggesting that the neddylation pathway plays an important role in SG assembly.

## Abstract (web summary)

Stress granules that form in response to stress contain translationally stalled mRNPs and play important roles in cellular homeostasis. Here the authors implicate SRSF3 neddylation as an important factor in the formation of stress granules in response to arsenite exposure.

## Graphical abstract

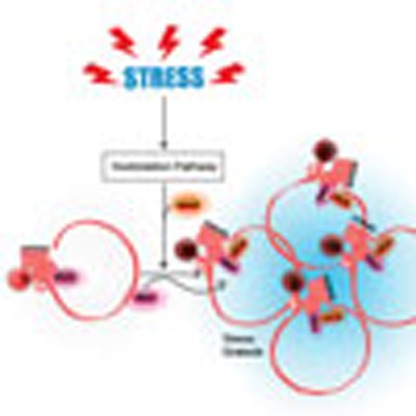

Stress granules (SGs) are non-membranous, cytoplasmic aggregates at which translationally stalled messenger ribonucleoprotein (mRNP) complexes are localized in response to various cellular stresses^1^. In cells exposed to adverse conditions, activation of the integrated stress response (ISR) leads to translational arrest, polysome disassembly and SG assembly^2^,^3^. By these mechanisms, the translation of mRNAs encoding housekeeping genes is repressed while translation of mRNAs encoding cytoprotective stress-responsive genes is preserved to enhance cell survival^4^. The signature constituents of SGs are non-canonical 48S preinitiation complexes harbouring non-translating mRNAs bound to small ribosomal proteins, 5′-cap (7-methyl guanosine, m^7^G) proximal initiation factors eIF4E, eIF4G, eIF4A, eIF3s and poly(A)-binding protein (PABP)^5^,^6^. SGs also contain numerous RNA-binding proteins (RBPs) that regulate mRNA translation (for example, TIA-1, TIAR, serine/arginine (SR)-rich splicing factor 3 (SRSF3), hnRNPs, TDP-43) and decay (for example, Argonautes and XRN1), as well as signal transducers (for example, TRAF2, G3BP1, RACK1 and TORC1) that modulate various cellular events such as cell growth and apoptosis^7^,^8^,^9^,^10^,^11^,^12^,^13^.

Several signalling pathways and their associated post-translational protein modifications have been shown to modulate SG assembly and disassembly. The phosphorylation of eIF2α through the ISR is a key initial step to stimulate SG assembly, although inhibition of eIF4A using drugs or lipid mediators have been reported to initiate SG assembly independently of phospho-eIF2α^14^,^15^. In response to stress, stress-responsive serine/threonine kinases (heme-regulated initiation factor 2α kinase (HRI); protein kinase RNA-activated (PKR); PKR-like endoplasmic reticulum (ER) kinase (PERK); general control non-derepressible 2 (GCN2) are auto-activated and phosphorylate eIF2α at Serine 51, leading to reduced levels of the eIF2–GTP–tRNAi^Met^ ternary complex that causes the inhibition of translation initiation that precedes polysome disassembly and SG assembly^4^,^16^. Phosphorylation of Ras-Gap Binding protein 3 (G3BP) at Serine 149 has been reported to regulate SG assembly^17^,^18^. During heat shock, focal adhesion kinase (FAK) also modulates SG assembly through targeting growth factor receptor-bound protein 7 (Grb7). Dual specificity tyrosine-phosphorylation-regulated kinase 3 (DYRK3) has recently shown to modulate SG dynamics through possibly targeting RBPs and proteins downstream of mTORC1 signalling^19^. *O*-GlcNAc modification of ribosomal proteins is known to regulate SG aggregation but not stress-induced translation inhibition and polysome disassembly^20^. Stress-responsive poly(ADP) ribosylation of SG components has also been implicated in SG aggregation via a potential scaffolding function^21^. Although relevant targets and associated mechanisms are unknown, ubiquitination and acetylation likely have important roles in SG assembly^22^. These previous studies suggest that multiple signalling pathways and related molecular targets are crucial to coordinate SG dynamics in cells exposed to various stresses.

NEDD8 (neural precursor cell expressed developmentally downregulated protein 8) is a small ubiquitin-like protein (UBL) that is covalently conjugated to Lys residues on protein substrates in a manner similar to ubiquitin. The NEDD8 conjugation system consists of a single E1-activating enzyme (NEDD8-activating E1 enzyme (NAE)), a heterodimer of amyloid-β precursor protein-binding protein 1 (APPBP1) and ubiquitin-activating enzyme 3 (UBA3), and two E2s, UBE2M (also known as UBC12) and UBE2F. NEDD8-specific E3 ligases are not well-understood and all currently reported E3s can also function in the ubiquitination system^23^. Neddylation primarily targets Cullin components of Cullin-RING Ligases (CRLs), although there are other known targets of Nedd8, including p53 and Histone H4 (refs 24, 25, 26).

In the present study, we show that protein neddylation is a modulator of SG assembly. We show that knockdown or inhibition of key components of the neddylation pathway impairs stress-induced polysome disassembly and SG assembly. Proteomic analysis utilizing *in vivo* biotinylation identifies translation factors, RBPs and ribosomal proteins as potential targets for neddylation. Because SRSF3 (also known as SRp20) was recently reported to be required for SG assembly^7^, we focused our attention on this target. We find that SRSF3 is neddylated on Lys85 in response to arsenite-induced oxidative stress and that a non-neddylatable SRSF3 (K85R) mutant is impaired in interacting with 5′-cap proximal translation initiation factors and the promotion SG assembly. Altogether, these results suggest that neddylation plays a critical role in the SG assembly, and that the neddylation of SRSF3 is at least one important event required for SG aggregation.

## Results

### Neddylation pathway regulates SG assembly

**Figure 1:**
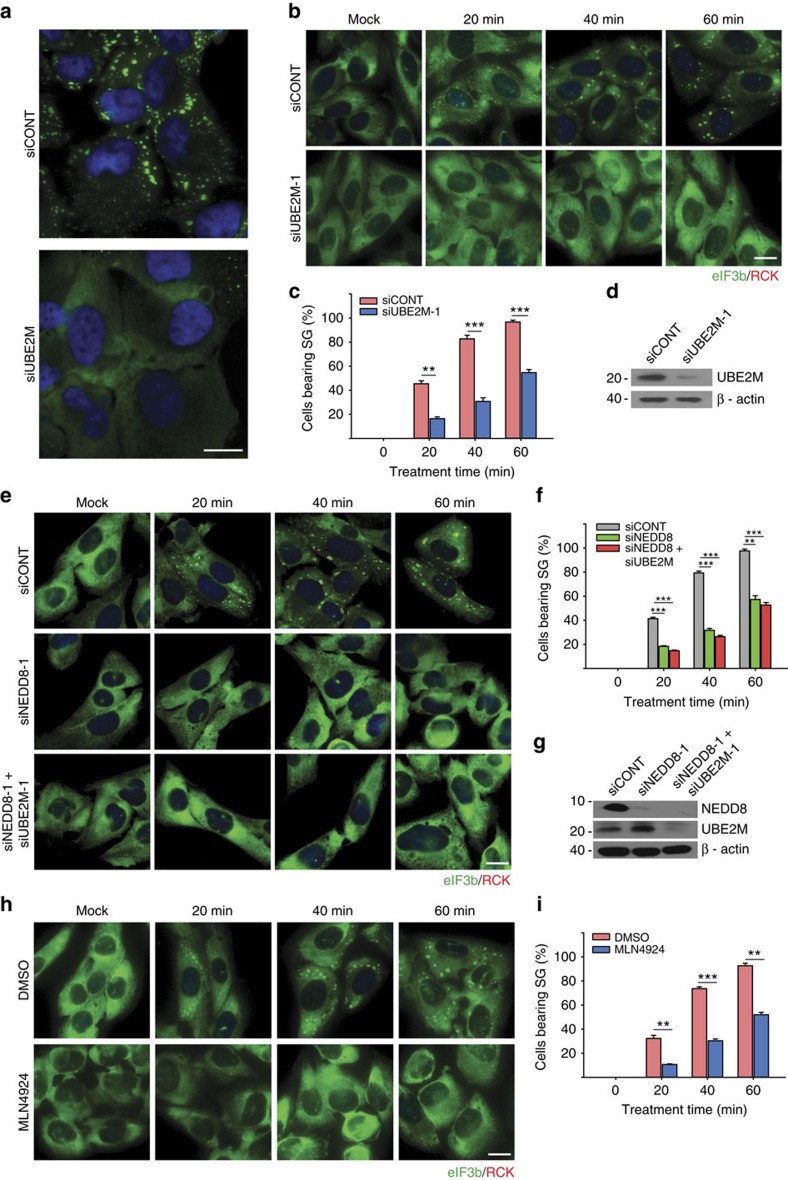
Neddylation pathway regulates SG assembly. (**a**) Images from siRNA screen plates with RDG3 stable cells showing knockdown effect of UBE2M on SG assembly. (**b**) U2OS cells transfected with siCONT or siUBE2M for 72 h were cultured in the absence or presence of arsenite (0.2 mM) for indicated time points, and then immunostained against SG marker eIF3b (green), PB marker RCK (red). Nuclei (Blue) are counterstained with bisbenzamide. (**c**) Bar graph representing the percentage of cells bearing SGs. Error bars indicate s.e.m. (*n*=4). **P<0.01, ***P<0.001, Student's *t*-test. (**d**) UBE2M knockdown efficiency was assessed using western blot analysis. (**e**) U2OS cells transfected with siCONT, siNEDD8 or mixture of siNEDD8 and siUBE2M were treated with 0.2 mM arsenite for indicated time points prior to processing for immunofluorescence microscopy using anti-eIF3b and anti-RCK antibodies. (**f**) Statistical graph showing percentage of cells bearing SGs. Error bars indicate s.e.m. (*n*=4). **P<0.01, ***P<0.001, Student's *t*-test. (**g**) Western analysis for knockdown efficiency of NEDD8 and UBE2M. (**h**) U2OS cells pretreated with DMSO or 1 μM ML4924 for 18 h were treated with 0.2 mM arsenite for indicated time points and stained against eIF3b and RCK. (**i**) Statistical data showing percentage of cells bearing SGs. Error bars indicate s.e.m. (*n*=3). **P<0.01, ***P<0.001, Student's *t*-test. Scale bar, 10 μm.

In our previous RNAi screen (∼7,300 genes) designed to identify genes whose expression is required for arsenite-induced SG assembly, the E2 conjugating enzyme UBE2M was a ‘hit'^20^. We confirmed that UBE2M knockdown significantly impairs the arsenite-induced SG assembly (Fig. 1a). To confirm this result, we knocked down UBE2M with different siRNA sequences (siUBE2M-1, siUBE2M-2) and monitored SG assembly kinetics using eIF3b as a SG marker (Fig. 1b,c; Supplementary Fig. 1a–c). The time-course experiment shows that cells treated with siUBE2M display significant defect in SG assembly under arsenite stress, compared with the cells treated with control siRNA (siCONT). The siUBE2M effectively depleted endogenous UBE2M expression as shown in western blot analysis (Fig. 1d).

Since UBE2M mediates protein neddylation, we hypothesized that the neddylation pathway might regulate SG assembly. To test this, we performed NEDD8 knockdown experiments using two different siRNAs (siNEDD8-1, siNEDD8-2). As shown in Fig. 1e–g and Supplementary Fig. 1, cells depleted of NEDD8 protein have a significant defect in eIF3b-positive SG assembly, similar to UBE2M knockdown. In addition, knockdown of both UBE2M and NEDD8 by combining siUBE2M and siNEDD8 shows a similar effect on the inhibition of SG formation (Fig. 1e–g). The inhibitory effect on SG assembly was confirmed by using different SG markers including TIA-1, eIF4G, G3BP or *O*-GlcNAc (Supplementary Fig. 2).

To further explore the impact of neddylation on SG assembly, we utilized MLN4924 (pevonedistat), a small molecule inhibitor of NAE, to block the cellular neddylation pathway^27^. In a dose–response analysis (Supplementary Fig. 3), inhibition of SG assembly was observed at a minimum concentration of 1 μM. A time-course analysis at this concentration revealed MLN4924-mediated inhibition of SG assembly was similar in magnitude to that observed following UBE2M and NEDD8 knockdown (Fig. 1h,i). These results were confirmed using HeLa and U2OS-derived EGFP–G3BP stable cell lines (Supplementary Fig. 4).

Arsenite-induced phosphorylation of eIF2α causes translational arrest, polysome disassembly and SG assembly^2^. We used sucrose gradient analysis to compare polysome profiles in cells expressing reduced levels of UBE2M and NEDD8. In siCONT-transfected cells, arsenite-induced translational arrest results in the collapse of polysome profiles and accumulation of monosomes and individual ribosomal subunits (Supplementary Fig. 5a, upper panels). Knockdown of both NEDD8 and UBE2M modestly increases the accumulation of non-translating ribosomal peaks but largely have no effect on polysomal peaks in the absence of stress (Supplementary Fig. 5a, lower left panel). In contrast, arsenite-induced polysome disassembly is partially inhibited in stressed cells with reduced NEDD8 and UBE2M expression (Supplementary Fig. 5a, lower right panel). The phosphorylation of eIF2α is indicative of polysome collapse and translation repression^28^. Interestingly, the basal levels of eIF2α phosphorylation in a non-stressed condition are marginally increased in NEDD8 or UBE2M knockdown cells that are likely reflected by marginally increased 80S monosome peaks. However, this marginal increase of eIF2α phosphorylation is not sufficient to induce polysome disassembly and SG assembly (Supplementary Fig. 5b,c). The extent of arsenite-induced phosphorylation of eIF2α in NEDD8 or UBE2M knockdown cells are comparable to that of control knockdown cells, suggesting that the neddylation pathway acts downstream of eIF2α phosphorylation (Supplementary Fig. 5c). Similarly, a time-course analysis of polysome profiles in cells treated with or without MLN4924 reveals a delay in arsenite-induced polysome disassembly (Supplementary Fig. 5d). These results argue that neddylation promotes polysome disassembly after ISR-induced translational arrest.

### UBE2M and NEDD8 are integral components of SG

**Figure 2:**
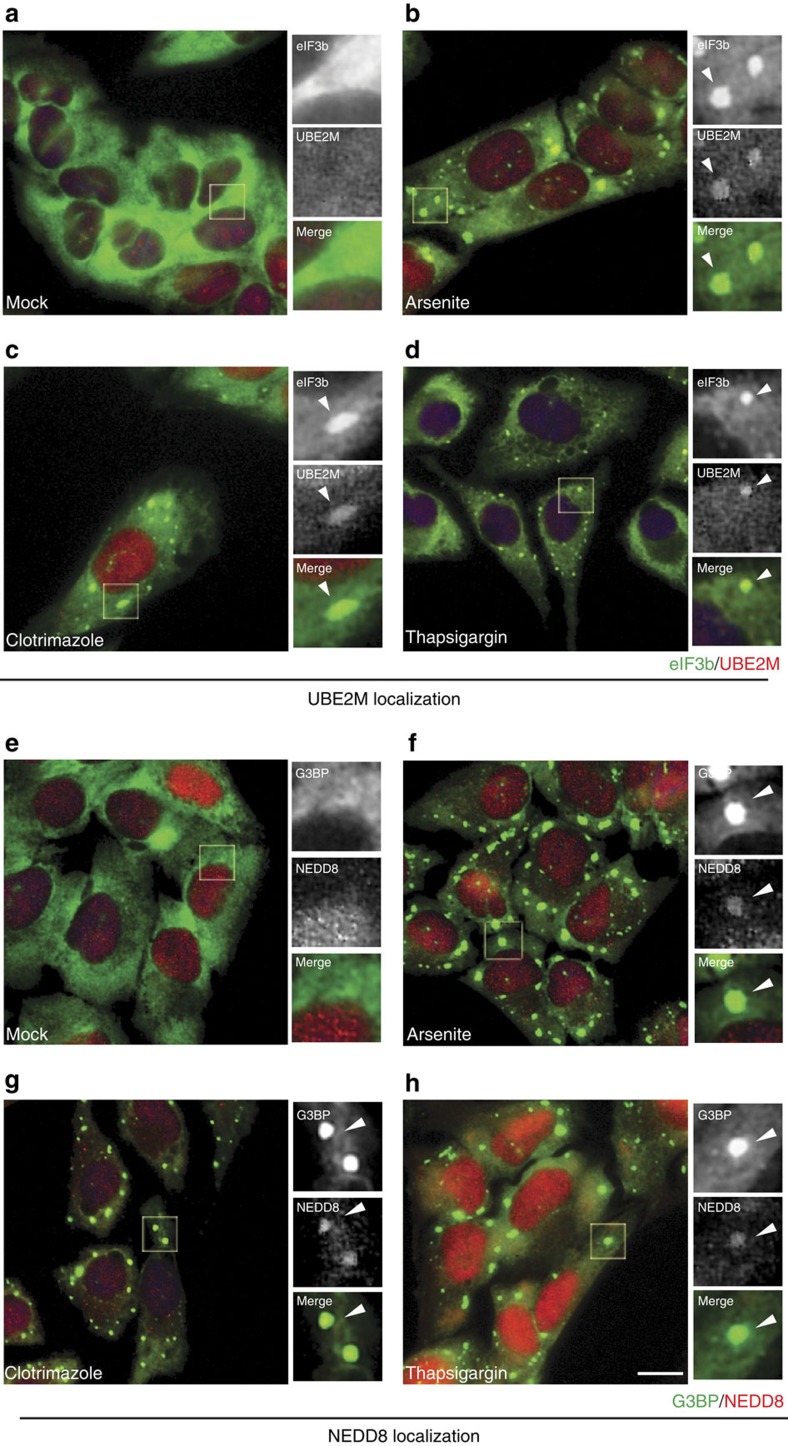
UBE2M and NEDD8 are integral components of SGs. (**a–d**) UBE2M is a component of SGs. U2OS cells grown on coverslips were either (**a**) untreated (mock) or treated with (**b**) 0.5 mM arsenite (**c**) 20 μM clotrimazole (**d**) 1 μM thapsigargin for 1 h. Samples were processed and immunostained against eIF3b (green) and UBE2M (red). (**e**–**h**) NEDD8 is a component of SGs. U2OS cells either (**e**) untreated or treated with (**f**) 0.5 mM arsenite (**g**) 20 μM clotrimazole (**h**) 1 μM thapsigargin for 1 h were processed and immunostained against G3BP (green) and NEDD8 (red). Boxed regions are enlarged as both merged and separate channel views. Arrowhead indicates SG. Scale bar, 10 μm.

Because altered expression of SG components generally affects SG dynamics, we next examined whether UBE2M and NEDD8 are present in SGs induced with arsenite (oxidative stress), clotrimazole (mitochondrial stress) or thapsigargin (ER stress) by employing indirect immunofluorescence microscopy using antibodies against UBE2M and NEDD8. In unstressed cells, UBE2M is diffusely distributed throughout the cell (Fig. 2a), while NEDD8 is concentrated in the nucleus (Fig. 2e). In stressed cells, spots of both UBE2M and NEDD8 became visible in SGs that are positive with eIF3b (Fig. 2b–d) and G3BP-containing SGs (Fig. 2f–h), respectively. Thus, we conclude that UBE2M and NEDD8 are integral components of SGs. Knockdown of NEDD8 or UBE2M also strongly inhibited clotrimazole or thapsigargin-induced SG formation (Supplementary Fig. 6), suggesting that the role of the neddylation system is not limited to the arsenite-induced stress.

### Inhibition of the neddylation does not affect PB assembly

P-bodies (PB) are a second class of cytoplasmic RNA granule whose composition and function are distinct from SGs^29^,^30^. To test whether perturbation of the neddylation pathway affects the PB assembly, PBs were visualized using antibodies reactive with RCK (DDX6) and S6K1 in cells treated with control, UBE2M and NEDD8 targeting siRNAs. As shown in Supplementary Fig. 7, neither UBE2M nor NEDD8 are required for constitutive or arsenite-induced PB assembly. MLN4924 treatment also does not alter the kinetics of PB assembly (Fig. 1h, Supplementary Fig. 3). These results suggest that neddylation pathway is required for the efficient assembly of SG, but not PB.

### Proteomics identifies neddylated proteins

**Figure 3:**
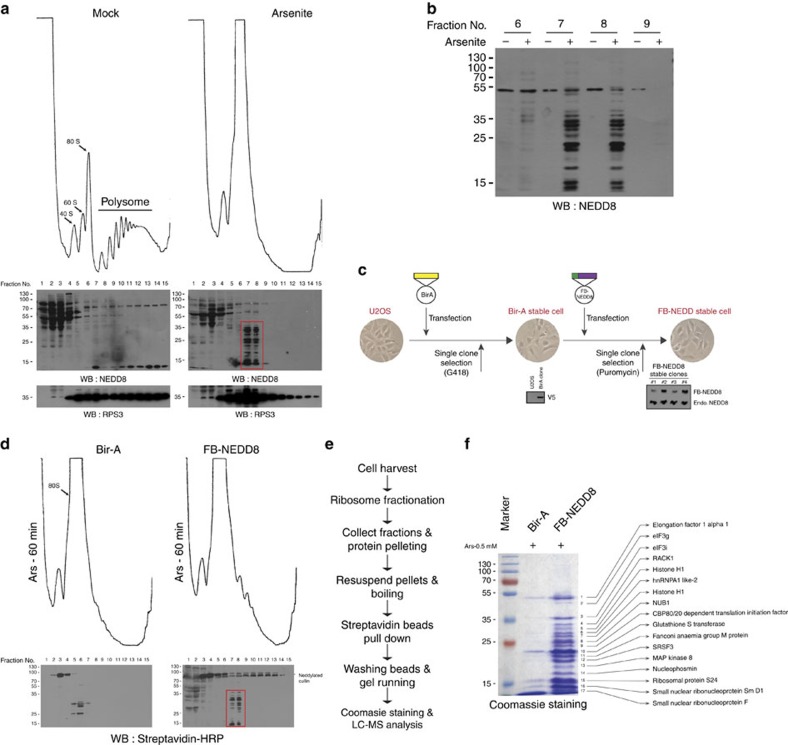
Identification of neddylated target proteins associated with translation machinery. (**a**) Arsenite-induced neddylated proteins are enriched in 80S monosome fractions. Mock or arsenite- (0.5 mM for 1 h) treated U2OS cells were subjected to polysome profiling analysis as described in Methods. A total of 15 fractions collected from sucrose gradient were acetone precipitated, centrifuged and the air-dried pellets were dissolved in SDS sample buffer. The samples were resolved in SDS–PAGE and blotted with anti-NEDD8 antibody. (**b**) Clear representation of accumulation of neddylated proteins in selected 80S monosome fractions under arsenite stress. (**c**) Schematic representation for generation of Flag–biopeptide (FB)-tagged NEDD8 stable cell line (see Methods). (**d**) Identification of arsenite-induced neddylated proteins in FB–NEDD8 stable cells. U2OS cells stably expressing Bir-A or FB–NEDD8 treated with arsenite (0.5 mM) for 1 h were lysed and subjected to polysome fractionation using sucrose gradients (17–50%). A total of 15 fractions were collected, acetone precipitated, air-dried and resolved in SDS–PAGE gel. The membranes were blocked in normal horse serum for 1 h before being blotted directly against streptavidin–HRP to detect NEDD8 conjugates. Bir-A stable cell line was used as control. (**e**) Brief procedure to isolate NEDD8 conjugates for MS analysis. (**f**) Isolation and immunopurification of stress-induced neddylated proteins. Stably expressing Bir-A and FB–NEDD8 cells treated with 0.5 mM arsenite for 1 h were subjected to sucrose gradient fractionation, and fraction No. 7 and 8 were collected, acetone precipitated and FB–NEDD8-conjugated proteins were affinity-purified using streptavidin beads. The precipitates were resolved in 12% SDS–PAGE and stained with Coomassie blue. A total of 17 distinct bands were identified and analysed through LC-MS. One representative identified protein from each band is shown.

In light of the above findings, we sought to identify proteins whose neddylation is required for SG assembly. To identify specific neddylated proteins involved in SG assembly, we first analysed sucrose gradient fractions obtained from control versus arsenite-treated cells (Fig. 3a). Interestingly, western blotting with an anti-NEDD8 antibody reveals the arsenite-induced neddylation of low-molecular-mass (10–40 kDa) proteins that sediment together with monosomes and untranslated mRNPs (Fig. 3a boxed region and Fig. 3b for western blotting with selected fractions). To affinity-purify those proteins, we employed an *in vivo* biotinylation system^31^ that has been successfully used in previous studies^32^. For the first step, bacterial biotin ligase (Bir-A) was stably expressed in U2OS cells and clonal selection was performed using G418 (Fig. 3c). Next, Flag–biopeptide- (FB-) tagged NEDD8 was stably expressed in Bir-A stable cells and clonal selection was performed using puromycin (see Methods). Of the four clones selected, we chose clone #2 which expresses a low level of FB–NEDD8 compared with endogenous NEDD8 for affinity purification (Fig. 3c, right western blot panel). The morphology and growth phenotype of FB–NEDD8 stable cells are similar to those of parental U2OS (Fig. 3c). More importantly, the level of arsenite-induced SG assembly in FB–NEDD8 stable cells is similar to that of U2OS parental cells (Supplementary Fig. 8). Western blotting of arsenite-treated ribosomal fraction samples with streptavidin–HRP conjugate displays similar pattern to that of immunoblot detected with NEDD8 antibody (compare boxed regions in Fig. 3a,d). These fractions were precipitated with acetone, boiled in 1% SDS to disrupt protein or mRNP complexes completely, then diluted and affinity-purified with streptavidin beads (Fig. 3e, see Methods for details). Neddylated proteins resolved on PAGE were revealed by staining with Coomasie blue (Fig. 3f); A total of 17 distinct bands were excised and subjected to mass-spectrometry (see Methods), which identified a large number of ribosomal proteins that are previously known neddylation targets^33^. Interestingly, eukaryotic translation initiation (eIF2α, eIF3g, eIF3m, eIF3i, eIF3h, eIF4AII, eIF6, CTIF) and elongation (eEF1α) factors were identified—many of these proteins are previously identified but unconfirmed neddylation targets^34^. In addition, many heterogeneous nuclear ribonucleoproteins (hnRNPs) and two SRSFs (SRSF1, SRSF3) that function in a wide range of RNA processing and regulation events were identified. The ribosome-associated protein RACK1 that was identified as an *O*-GlcNAc modification target is also in the list (Supplementary Data 1). Because translation factors and RBPs are well-known regulators of SG assembly, these findings suggest that neddylation of these proteins might promote SG assembly.

It was interesting for us to find SRSF3 from the proteomic study, because we recently reported that SRSF3 is a novel and necessary SG component and when depleted SGs are potently disrupted^7^,^20^. The knockdown effect of SRSF3 on SG assembly was confirmed with different siRNAs in U2OS cells (Supplementary Fig. 9a–c). Endogenous and Flag-tagged SRSF3 staining also revealed its presence in SGs (Supplementary Fig. 9d,e). Based on this evidence, we selected SRSF3 to explore the possible mechanistic link between the neddylation pathway and SG assembly.

### SRSF3 is neddylated in cells subjected to arsenite stress

**Figure 4:**
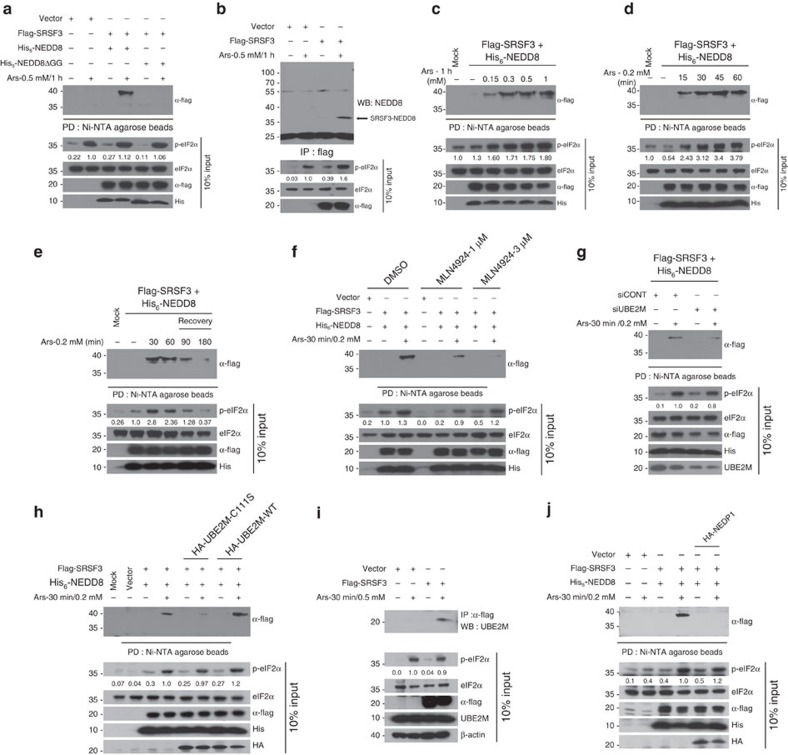
SRSF3 is a novel target for stress-induced neddylation *in vivo*. (**a**) HEK293T cells were transiently co-transfected with indicated plasmids. After 36 h, cells were treated with either mock or arsenite, lysed under denaturing condition and affinity-purified using Ni^2+^-NTA agarose beads (see Methods). The precipitates were then blotted against anti-Flag antibody. (**b**) HEK293T cells transfected with empty vector or Flag–SRSF3 were treated with arsenite, lysed under denaturing condition (1% SDS) and immunoprecipitated using Flag beads (see Methods). (**c**) *In vivo* neddylation assay was carried out for cells treated with arsenite in a dose-dependent manner (0.15 to 1 mM) for 1 h. (**d**) *In vivo* neddylation assay was performed for cells treated with 0.2 mM arsenite at different time points (0–60 min) as indicated. (**e**) HEK293T cells transfected with indicated plasmids were treated with 0.2 mM arsenite in lane 3 and 4 for 30 min and 60 min, respectively. Cells in lanes 5 and 6 were treated with 0.2 mM arsenite for 60 min and then allowed to recover from stress by changing with fresh medium for 90 min (lane 5) and 180 min (lane 6) and subjected to neddylation assay. (**f**) NAE inhibitor MLN4924 significantly attenuates SRSF3 neddylation. HEK293T cells pretreated with DMSO, MLN4924 (1 μM) for 18 h or MLN4924 (3 μM) for 1 h prior to arsenite treatment were subjected to neddylation assay. (**g**) SiRNA mediated knockdown of UBE2M reduces SRSF3 neddylation. *In vivo* neddylation assay was performed for HEK293T cells transfected with siCONT or siUBE2M under arsenite stress. (**h**) Ectopic expression of UBE2M-C111S displays dominant negative effect on SRSF3 neddylation. HEK293T cells co-transfected with indicated plasmids were treated with arsenite and subjected to neddylation assay. (**i**) UBE2M interacts with SRSF3 under arsenite stress. Empty vector or Flag–SRSF3 transfected cells were treated with arsenite, lysed in IP buffer and immunoprecipitated using Flag agarose beads. The precipitates were subjected to western analysis using anti-UBE2M antibody. (**j**) NEDP1 overexpression inhibits SRSF3 neddylation. HEK293T cells transfected with indicated plasmids were treated with arsenite and affinity purified using Ni^2+^-NTA agarose beads.

To confirm whether SRSF3 is indeed neddylated, we undertook a neddylation assay under denaturing conditions using HEK293T cells^35^. Flag–SRSF3 was transfected with either His-NEDD8 or His-NEDD8ΔGG (a non-conjugatable NEDD8), then mock or arsenite-treated cells were harvested under denaturing conditions, and affinity pull-down assay was performed followed by western blotting analysis (see Methods). We detected neddylated Flag–SRSF3 at ∼40 kDa consistent with di-neddylation in cells treated with arsenite (Fig. 4a). None of the experiments utilizing empty vector or His-NEDD8ΔGG display the corresponding band, suggesting that SRSF3 is conjugated with NEDD8 in response to stress. We also confirmed that endogenous NEDD8 is conjugated to Flag–SRSF3 under arsenite stress (Fig. 4b). The stress-induced neddylation of SRSF3 is dose- and time-dependent (Fig. 4c,d) and the effect of arsenite is reversible; the neddylated species disappear on recovery from arsenite (Fig. 4e). The neddylation of SRSF3 was dependent on neddylating enzymes NAE1 and UBE2M, as inhibition of NAE1 by treatment of MLN4924, or knockdown of UBE2M effectively inhibited the formation of Nedd8–SRSF3 (Fig. 4f,g).

Overexpressing a catalytically inactive UBE2M (C111S), but not the wild type (WT), led to a decrease in the NEDD8–SRSF3 species, implying its dominant negative effect (Fig. 4h). Co-immunoprecipitation analysis suggests that Flag–SRSF3 interacts with UBE2M, particularly when cells were treated with arsenite (Fig. 4i). Finally, to further confirm if the stress-induced upshifted Flag–SRSF3 is indeed NEDD8-conjugated form, NEDP1 (also known as DEN1 or SENP8), a NEDD8-specific Cys protease, was co-expressed. As shown in Fig. 4j, while expression of Flag–SRSF3 in the presence of His-NEDD8 results in appearance of modified Flag–SRSF3 species, these species were not detected when haemagglutanin (HA)–NEDP1 is co-expressed (Fig. 4j, compare lane 4 and 6). We also found that the SG formation is effectively inhibited when the HA–UBE2M mutant (C111S) or HA–NEDP1 is overexpressed (Supplementary Fig. 10), which is consistent with our model that the neddylation of SRSF3 is necessary for the SG formation. Collectively, these data strongly support that neddylation of SRSF3 is induced by arsenite stress through NAE–UBE2M axis of neddylation system and this may lead to SG formation.

### SRSF3 is neddylated at Lys85

**Figure 5:**
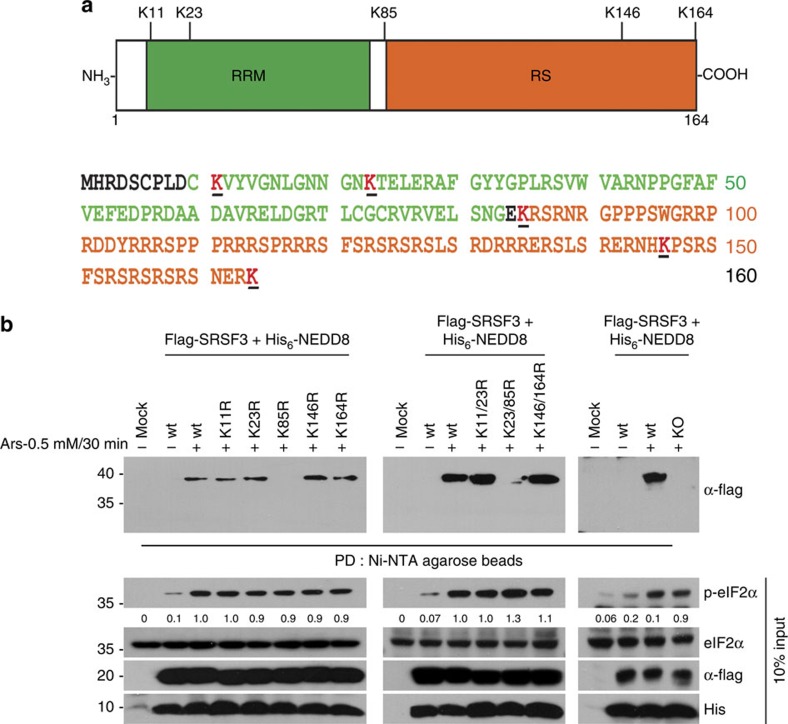
SRSF3 is neddylated at Lys85 under arsenite stress. (**a**) Schematic representation of SRSF3 protein domains and positions of lysine residue. SRSF3 contains 2 domains, one RRM and one RS domain and has 5 lysine residues positioned at 11, 23, 85, 146 and 164 (highlighted by underline). (**b**) SRSF3 is neddylated at Lys85 under arsenite stress. His-NEDD8 was co-transfected with empty vector or Flag–SRSF3 WT and series of single lysine mutant (11, 23, 85, 146, 164), double lysine mutant (11/23, 23/85, 146/164) or null mutant (all lysine mutant) as indicated and treated with 0.5 mM arsenite for 30 min. Cells were then lysed under denaturing condition, affinity-purified with Ni-NTA agarose beads and the precipitates were subjected to western analysis using anti-Flag antibody.

SRSF3 is the smallest member of the highly conserved SRSF family and is composed of one structured RNA recognition motif (RRM) at the N terminus and one disordered RS domain at the C terminus^36^. The protein is composed of 164 amino acids, about half of which belong to the RS domain (Fig. 5a). There are five lysine residues that may be targeted for neddylation: two reside in the RRM domain, two in the RS domain and one in between.

To identify residue(s) that are neddylated in response to arsenite, we constructed a series of K-R mutants (K11R, K23R, K85R, K146R, K164R; double mutants, K11/23R, K23/85R, K146/164R; all lysine mutant, KO). *In vivo* neddylation assays with these mutants revealed that mutants with K85R selectively lack arsenite-induced SRSF3 neddylation (Fig. 5b).

### SRSF3 K85R mutation impairs arsenite-induced SG assembly

**Figure 6:**
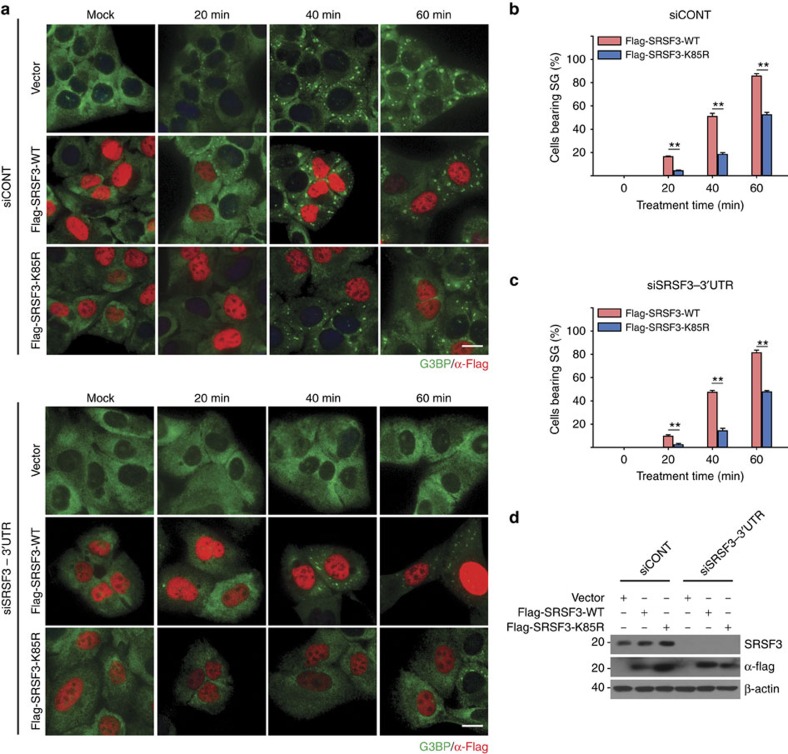
Flag–SRSF3-WT but not K85R mutant can rescue the SG assembly defect in SRSF3-depleted cells. (**a**) Stably expressing empty vector, Flag–SRSF3-WT or K85R mutant cells transfected with siCONT or siSRSF3-3′UTR were treated with 0.2 mM arsenite before processing for immunofluorescence microscopy using anti-G3BP and anti-Flag antibody. Percentage of cells bearing SGs in (**b**) siCONT-transfected cells and (**c**) siSRSF3-3′UTR-transfected cells are shown as bar graph. Error bars indicate s.e.m. (*n*=4). **P<0.01, Student's *t*-test. (**d**) Knockdown efficiency of endogenous SRSF3 and the expression Flag constructs were assessed by western blot analysis. Scale bar, 10 μm.

To test the physiological role of neddylation of SRSF3 at K85, we stably expressed either WT or SRSF3 (K85R) in U2OS cells and monitored the dynamics of SG assembly. Interestingly, we observed that Flag–SRSF3–K85R-expressing cells display a significant delay in SG assembly that phenocopies knockdown or inhibition of neddylation components, whereas Flag-SRSF3-WT cells exhibit normal SG assembly (Supplementary Fig. 11; note that the nuclei of Flag–SRSF3 transfected cells are stained red). To convincingly show the overexpression effects on SG assembly in Flag–SRSF3–WT or Flag–SRSF3–K85R stable cells, we depleted endogenous SRSF3 utilizing siRNA targeting 3′ untranslated region (UTR) of SRSF3 mRNA and monitored SG dynamics in cells expressing WT or K85R protein over time. In siCONT-transfected cells, the results of SG assembly dynamics are similar to those in Supplementary Fig. 11 (Fig. 6a,b). In siSRSF3-3′UTR transfected cells, SG assembly is normally rescued only in cells expressing Flag–SRSF3–WT, but not in cells expressing Flag–SRSF3–K85R that displays a slightly more inhibitory effect on SG assembly when compared with those transfected with siCONT (Fig. 6b,c). Knockdown of endogenous SRSF3 and overexpressed exogenous Flag–SRSF3–WT and –K85R proteins were confirmed with western analysis (Fig. 6d). Interestingly, the impairment of Flag–SRSF3–K85R localization to SG was not observed under different stresses such as clotrimazole and thapsigargin. Moreover, the neddylation of Flag–SRSF3 could not be detected under those stresses, suggesting that the promotion of SG formation through SRSF3 neddylation is arsenite-specific event (Supplementary Fig. 12). We also checked the effects of other single or double lysine mutants on SG assembly and found that those are comparable to that of WT except that K146/164R mutations led to marginal inhibitory effects (Supplementary Fig. 13). The slight inhibitory effect of K146/164R mutant is possibly due to its location in unstructured RS domain, which is correlated with recent findings that unstructured peptide sequences such as prion-like or low-complexity domains are crucial for mRNP aggregation^4^.

Given that knockdown of SRSF3 abrogate both SG and PB assembly, we tested whether expressing the SRSF3–K85R mutant has an impact on PB assembly using S6K1 as a PB marker. As shown in Supplementary Fig. 14, both WT and K85R mutant display a comparable PB assembly pattern in the presence or absence of arsenite, indicating that the arsenite-induced SRSF3 neddylation selectively modulates SG assembly.

### SRSF3 K85R has defects in association with SG components

**Figure 7:**
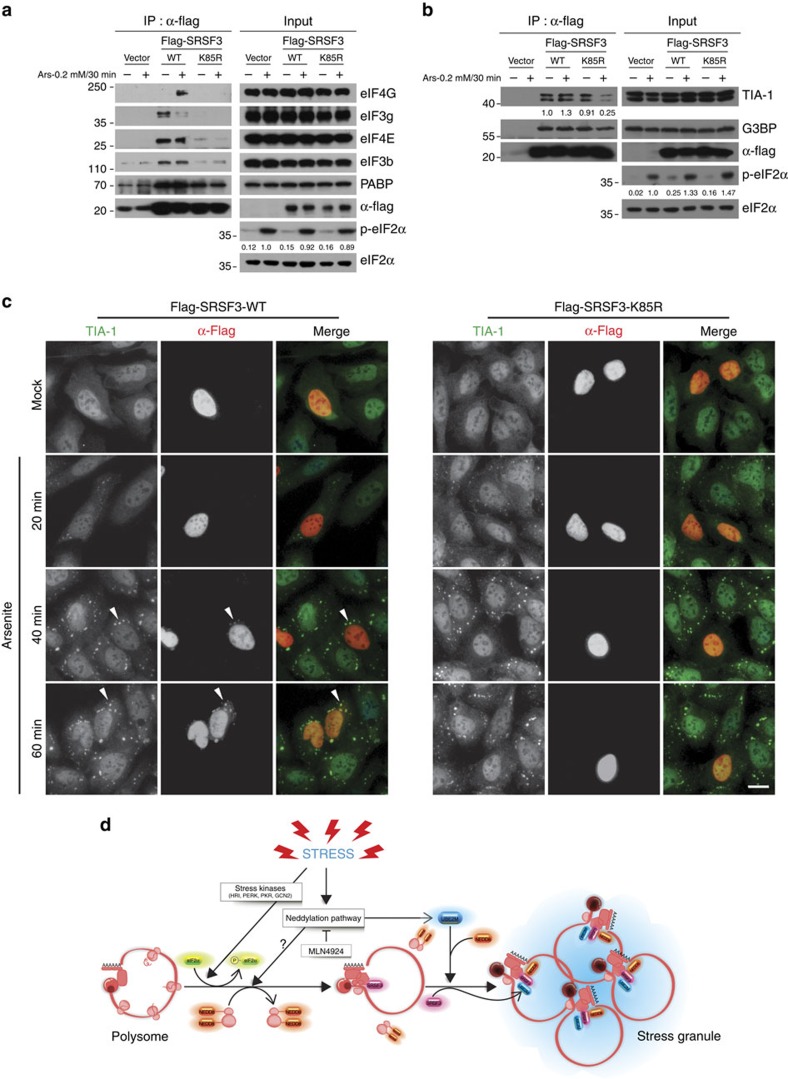
SRSF3–K85R displays defects in association with SG components. (**a**) Empty vector, Flag–SRSF3-WT or K85R mutant transfected cells were treated with arsenite for 30 min, lysed in IP buffer and immunoprecipitated using Flag agarose beads. The precipitates were subjected to western analysis using antibodies against SG proteins. (**b**) SRSF3–K85R has a defect in associating with TIA-1 under arsenite stress. Immunoprecipitation was carried out as described in **a** and the precipitates were blotted against TIA-1 and G3BP. (**c**) U2OS cells transiently transfected with Flag–SRSF3-WT or K85R mutant were treated with either mock or 0.2 mM arsenite and subjected to immunofluorescence microscopy. Recruitment of TIA-1 into SGs was assessed by co-staining with anti-TIA-1 and anti-Flag antibodies. Arrowhead indicates SG. (**d**) Model diagram of SG assembly mediated by neddylation pathway. During stressful condition, stress kinases (HRI, PKR, PERK, and GCN2) are activated and phosphorylate eIF2α to initiate abortive translation initiation and polysome disassembly. Subsequently, neddylation pathway possibly targets ribosomal proteins to promote polysome disassembly. Finally, neddylation of SRSF3 promotes SG aggregation through interaction with initiation factors such as eIF4G, eIF3s and SG assembly factor TIA-1. Scale bar, 10 μm.

Recently, we reported that SRSF3 functions as a translation repressor of PDCD4 mRNA by binding its 5′-UTR region^37^. In line with this study, we identified interacting proteins that are mechanistically relevant to its translational regulatory function using proteomic analysis of SRSF3 immunoprecipitates. Several translation factors including nuclear cap-binding protein (NCBP1), eukaryotic translation initiation factor 2A (EIF2A), eukaryotic translation initiation factor 2 subunit 3 (EIF2S3), eukaryotic initiation factor 4A-1 (EIF4A1) and polyadenylate-binding protein 1 (PABP1) were found to interact with SRSF3. To determine whether SRSF3 neddylation modulates the interaction with these factors, we compared the ability of WT and mutant (K85R) SRSF3 to immunoprecipitate translation factors from lysates prepared from cells cultured in the absence or presence of arsenite. Interestingly, while WT and the K85R mutant are associated with PABP at a similar level, the levels of eIF4E, eIF4G and eIF3 associated with the K85R mutant is noticeably lower compared with WT (Fig. 7a). It has been well-characterized that several proteins such as TIA-1 and G3BP1, which contain the aggregation prone domain, mediate the SG aggregation and overexpression of these causes abnormal SG formation^17^,^38^. Hence, we assessed if the SRSF3–K85R mutant also have defects in associating with these proteins under mock or arsenite stressed condition. As shown in Fig. 7b, association of TIA-1 with the K85R mutant is much reduced compared with the WT under arsenite stress while G3BP1 displays a comparable association compared with WT. The association defect seen only in the arsenite condition may imply that mRNP remodelling for the SG aggregation is defective with the K85R mutant. Consistently, the recruitment of TIA-1 in SG under arsenite stress is strongly abrogated in cells expressing the K85R mutant, whereas the SRSF3-WT-expressing cells display TIA-1-positive SGs that is colocalized with Flag–SRSF3–WT (Fig. 7c). These results indeed support that the neddylation of SRSF3 is critical for interacting with and facilitating the recruitment of TIA-1 to SG.

Finally, because inhibiting neddylation delays stress-induced polysome disassembly (Supplementary Fig. 5), we wanted to determine to what extent the SRSF3 neddylation is responsible for this phenotype. Polysome profiles of cells expressing the WT or the K85R SRSF3 showed similar pattern (Supplementary Fig. 15a). This result suggests that delayed polysome disassembly shown in the cells defective in neddylation is not due to an inhibition of the SRSF3 neddylation, rather it could be caused by neddylation of unknown proteins. Because eIF2α phosphorylation is prerequisite for arsenite-induced polysome disassembly, we additionally tested whether knockdown of HRI, the kinase responsible for the arsenite-induced eIF2α phosphorylation^28^, dampen the neddylation of SRSF3. We found that knockdown of HRI effectively inhibited the eIF2α phosphorylation under arsenite as previously reported, but this does not affect the arsenite-induced neddylation of SRSF3 (Supplementary Fig. 15b). This observation suggests that the neddylation of SRSF3 at K85 is important for the aggregation process of SG, which is a separate event from the eIF2α phosphorylation and polysome disassembly.

## Discussion

Here we report that neddylation of SRSF3 is required for it to promote SG assembly. Our results reveal that: (1) SRSF3 neddylation is induced by arsenite stress, (2) the neddylation and deneddylation of SRSF3 following the application and removal of stress stimuli correlates with the assembly and disassembly of SGs (Fig. 4c–e), (3) MLN4924 inhibits stress-induced neddylation of SRSF3 in a dose-dependent manner, (4) knockdown of UBE2M dampens stress-induced neddylation of SRSF3, (5) UBE2M and NEDD8 are integral components of SGs, (6) SRSF3 associates with UBE2M in response to arsenite, and (7) SRSF3 neddylation at Lys85 promotes SG assembly likely at the aggregation stage. The latter conclusion is based on the observation that the K85R-expressing cells show similar stress-induced polysome disassembly when compared with WT (Supplementary Fig. 15), whereas blocking neddylation pathway inhibits polysome disassembly. This implies that neddylation of other targets (for example, ribosomal proteins, eEF1α) might be necessary for the disassembly of polysome (see working model in Fig. 7d).

It is unclear whether the neddylation functions upstream or downstream of the eIF2α phosphorylation, a well-established, critical event in the SG assembly. We found that inhibition of the neddylation pathway does not affect the arsenite-induced eIF2α phosphorylation, indicating that it acts independently and might have roles in polysome disassembly and/or mRNP remodelling for SG aggregation. In fact, the polysome profiling analyses revealed that arsenite-induced ribosome run-off process is significantly reduced in the cells defective in neddylation (Supplementary Fig. 5), similar to the phenotypes seen in the cells deficient in translation initiation factor 5A (eIF5A)^39^. This suggests that the neddylation pathway may be necessary for ribosome transit under normal and/or stress condition. Indeed, our proteomic screen identifies elongation factor 1-alpha (eEF1α) as a candidate for neddylation. It would be interesting in future research to investigate functional link between neddylation and translation elongation process.

We do understand the concern of possible artificial conjugation of overexpressed Nedd8 (ref. 40). Our analyses show that expression of FB–Nedd8 is lower than the endogenous level of Nedd8 (Fig. 3c), and that expressing FB–Nedd8 does not noticeably alter the polysome profile (Fig. 3d).

Also, FB–Nedd8-conjugated proteins are visible in the non-translating monosome fractions by streptavidin–HRP western blot and the pattern is similar to that of anti-Nedd8 western blot (compare Fig. 3a,d boxed regions). Most importantly, we showed that Flag–SRSF3 is conjugated with endogenous NEDD8 under arsenite stress (Fig. 4b) and SRSF3 neddylation is effectively inhibited by MLN4924 treatment (Fig. 4f), arguing that SRSF3 is a *bona fide* target^41^.

Unlike other post-translational modification pathways such as *O*-GlcNAcylation and ubiquitination that are dramatically increased on arsenite stress^42^,^43^, global upregulation of neddylation is not observed under these conditions. However, sucrose gradient ribosome fractionation uncovers likely stress-induced neddylated protein species sized 10–40 kDa that mostly reside in stalled 80S monosome fractions (Fig. 3a). The biotin–streptavidin pull-down strategy using those concentrated fractions allowed us to identify several novel neddylation targets that function in RNA metabolism, mostly in the translation process. Among the identified proteins, ribosomal proteins are most prevalent, consistent with previous findings^33^. Additional neddylated proteins that function in translation initiation (eIF2α, eIF4AII, components of eIF3 complex (i, g, h, m), and eIF6) and elongation (eEF1α) were discovered. EIF3g and eIF3i are strong hits from our previous RNAi screen and it is possible that neddylation of these proteins might play key roles in modulating translation in stressed cells. The ribosome-associated protein RACK1, a known *O*-GlcNAcylation target^20^, was also identified as a potential target of stress-induced neddylation. RACK1 has key roles related to ribosome activation and the cellular stress response. First, it associates with protein kinase C (PKC) and stimulates the joining of 40 and 60S subunits by promoting eIF6 phosphorylation, thereby activating translation^44^. Second, it serves as a scaffold protein required for stress-activation of JNK MAPK (SAPK) pathways, which promote apoptosis in response to genotoxic stresses which do not cause SG assembly^11^. In cells subjected to arsenite stress, RACK1 is sequestered in SGs, thereby inactivating the SAPK apoptotic response. Given these two separate observations and our findings here, we propose that RACK1 neddylation under arsenite stress might prevent monosome joining to inhibit global translation and inactivate the SAPK pathway.

Recently, several studies have identified pathological SGs related to many types of neuro-degenerative diseases such as Alzheimer's dementia, Multi-system proteinopathy (MSP), amyotropic lateral sclerosis (ALS) and frontotemporal lobar degeneration^45^,^46^. Most of these disease symptoms are likely mediated through mutated RBPs that produce stabilized SG-like aggregates, insoluble inclusion bodies, pathological fibril formation^8^ or defects of autophagic system, which is required for SG clearance during stress recovery^47^. Hence, unravelling the molecular mechanisms involved in the dynamic assembly and disassembly of SGs may lead to an improved understanding of the pathology of the SG-related neurological disorders. An interesting study has recently identified a pathogenic mutation in hnRNP A2B1 and A1 which potentially causes inherited MSP and ALS^8^. Notably, these mutations reside in a prion-like domain, which promote excessive SG aggregation and cytoplasmic inclusions. Our identification of hnRNPs in the proteomic screen for neddylation (Supplementary Data 1) could add another layer of complexity to the role of hnRNP aggregates in these disease syndromes. It will be important to determine the functional implications of hnRNP neddylation in the assembly of SGs.

Previous study showed that Ube1, a ubiquitin E1 enzyme, mediates conjugation of NEDD8 under diverse stress conditions^48^. Interestingly, treatment of PYR-41, an ubiquitin E1 enzyme inhibitor, strongly inhibited the neddylation of SRSF3 to a similar degree with MLN4924 (Supplementary Fig. 16a). Knockdown of Ube1 also dampen the arsenite-induced neddylation of Flag–SRSF3 (Supplementary Fig. 16b). To compare the effects of UBE2M-mediated canonical and Ube1-mediated atypical neddylation pathways on SG formation under arsenite stress, we transfected U2OS cells with siCONT, siUbe1 and siUBE2M and SGs were visualized using eIF3b, TIA-1 and G3BP. Unexpectedly, Ube1-knockdown cells display a slight decrease (∼20%) while UBE2M knockdown cells display an outstanding decrease (∼60%) in SG formation, suggesting that the canonical pathway is more essential for the SG formation (Supplementary Fig. 16c–f).

Consistent with a previous report that the K85 residue of SRSF3 is ubiquitinated^49^, we found that SRSF3 indeed is ubiquitinated in both conditions (Supplementary Fig. 16g). These observations suggest that the K85 residue of SRSF3 may be subjected to mixed modification of ubiquitin and NEDD8, or it is possible that distinct pools of ubiquitin and NEDD8-modified SRSF3 may exist under arsenite stress. Interestingly, a recent study found that NEDD8 can form mixed chain with ubiquitin where NEDD8 acts as a chain terminator^50^. Further studies are needed to define whether SRSF3 is modified with ubiquitin/nedd8 dipeptide chain and if so, what the physiological significance of this complex modification is.

The responsible E3 ligase for SRSF3 neddylation remains to be determined. So far, the best-characterized NEDD8 E3 ligases are RBX1 and RBX2, which work with UBE2M and UBE2F, respectively^51^,^52^. While knockdown of RBX2 had no effect on SG assembly kinetics, RBX1 knockdown produced a slight delay in SG assembly at early time points (Supplementary Fig. 17a–c), suggesting that RBX1 or RBX2 are not major players in stress-inducible SG assembly. Supporting this conclusion is the fact that; (1) neither these E3 ligases nor Cullin components were identified in our RNAi screen for SG assembly, and (2) RBX1 or RBX2 are not detected in SG (Supplementary Fig. 17d,e). Hence, we conclude that the inhibitory effect of SG formation on blocking neddylation is cullin-independent. Based on the observation that Flag–SRSF3 physically interacts with UBE2M (Fig. 4i), it is possible that UBE2M may act directly on SRSF3 for Nedd8 conjugation. Further studies are needed to validate this result or possibly identify additional E3 ligases.

## Methods

### Cell culture and transfection

U2OS (human osteosarcoma), HeLa and HEK293T cells were obtained from ATCC and maintained in DMEM medium (Welgene) supplemented with 10% inactivated FBS (Welgene), 1% (v/v) penicillin and streptomycin (Lonza) at 37 °C in 5% CO_2._ Transfection of siRNAs were performed using Lipofectamine 2000 (Invitrogen) at 40 nM final concentration, all siRNA sequences used in this study are listed in Supplementary Table 1. All DNA plasmids were transfected using either PEI (Polysciences) or Fugene 6 (Promega, Madison, WI) as per manufacture's protocol.

### Cloning

Human *NEDD8* cDNA was subcloned into pEFα1-FB vector. The human *SRSF3* cDNA was subcloned into pCI-Neo-Flag vector. The human *UBE2M*, *UBE2M-C111S* and *NEDP1* were subcloned into pcDNA3.1-HA vector. *SRSF3* single (11, 23, 85, 146, 164), double (11/23, 23/85, 146/164) and KO mutants (all lysine) were created by PCR-directed mutagenesis and subcloned into pCI-neo-Flag vector. Primers used for cloning are listed in Supplementary Table 2.

### Immunofluorescence analysis

Cells grown on coverslips were mock treated or treated with indicated drugs, rinsed twice with PBS (pH 7.4), fixed with paraformaldehyde for 15 min, permeabilized with cold methanol for 10 min and then blocked in 5% normal horse serum in PBS containing 0.02% sodium azide for 1 h. Primary antibodies diluted in blocking solution were added and incubated either at room temperature (RT) for 1 h or overnight at 4 °C. Cells were then washed with PBS (three times, 10 min each) and incubated with respective secondary antibodies (Jackson Immunoresearch ML grade) for 1 h at RT. After incubation, samples were washed thrice with PBS (10 min each) and mounted in polyvinyl medium. All images were taken using a Nikon Eclipse 80i fluorescence microscope, processed in Image J and compiled using Adobe Photoshop CS5. Knockdown or overexpression effects on SGs and PBs were assessed by quantifying the number of cells out of at least 100 cells from different fields as percentage.

### Immunoprecipitation

Cells were harvested and lysed in IP buffer (50 mM Tris-Cl (pH 7.5), 150 mM NaCl, 1 mM EDTA, 1% Trition X-100) supplemented with proteinase inhibitors 1 mM PMSF, 10 μg ml^−1^ aprotonin, 5 μg ml^−1^ leupeptin, 0.5 μg ml^−1^ pepstatin and 5 mM NaF on ice for 20 min, centrifuged at high speed for 15 min and the supernatants were collected in fresh tube. For immunoprecipitation, 1–2 mg lysate was incubated with 20–30 μl Flag agarose beads overnight at 4 °C. The resulting immunoprecipitates were washed at least three times in IP buffer, before boiling with SDS sample buffer. The resulting eluates were blotted against indicated antibodies.

### Immunoblot analysis

Cells were lysed in RIPA buffer (50 mM Tris-Cl (pH 8.0), 150 mM NaCl, 0.1% SDS, 1% NP-40, 1 mM EDTA, 1% Sodium deoxycholate, containing proteinase inhibitors 5 mM NaF, 1 mM PMSF) for 15 min in ice and centrifuged at 13,000 r.p.m. for 15 min. Proteins were quantified using Bradford reagent. Total proteins (20–50 μg) were subjected to SDS–PAGE, transferred to nitrocellulose membranes and detected with respective antibodies. western blot was performed using ECL detection system. Uncropped blots are shown in Supplementary Fig. 18. All antibodies used are listed in Supplementary Table 3.

### Polysome profiling analysis

U2OS cells grown at ∼90% confluency were treated with indicated time and concentration of sodium arsenite. After treatment, 10 μg ml^−1^ cycloheximide was added and incubated for 5 min at RT, washed with cold PBS, then lysed with 1 ml of polysome lysis buffer (20 mM HEPES pH 7.6, 5 mM MgCl_2_, 125 mM KCl, 1% NP-40, 2 mM DTT) supplemented with 100 μg ml^−1^ cycloheximide (Sigma), protease inhibitor cocktail (EDTA-free; Pierce) and RNAsin (Ambion) at cold room. Cell lysates were tumbled for 15 min at 4 °C and centrifuged at 13,000 r.p.m. for 15 min. The supernatants were fractionated in 17.5–50% linear sucrose gradients by ultracentrifugation (35,000 r.p.m. for 2 h 40 min) in a Beckman ultracentrifuge using SW40-Ti rotor. Gradients were eluted with a gradient fractionator (Brandel) and monitored with a UA-5 detector (ISCO). Fractions were acetone precipitated at −20 °C for overnight and processed for further analysis.

### Immunopurification of nedd8-modified proteins

U2OS cells stably expressing Bir-A or FB–NEDD8 (6 × 150-mm dish) treated with 0.5 mM arsenite for 1 h were harvested in polysome lysis buffer and subjected to sucrose gradient as described earlier. To purify nedd8-modified proteins, fraction numbers 7 and 8 (which are enriched with neddylated proteins) were pooled, acetone precipitated overnight at −20 °C, centrifuged at high speed for 15 min and the pellets were air-dried. Air-dried pellets were resuspended in 100 μl denaturing buffer (50 mM Tris-Cl pH 7.6, 2 mM EDTA, 1% SDS), boiled at 60 °C for 10 min and diluted to 1 ml using dilution buffer (50 mM Tris-Cl(pH 7.6), 2 mM EDTA, 150 mM NaCl, 0.1% NP-40) containing protease inhibitor cocktail, 5 mM NaF and 10 mM IAA (Sigma). Denatured total proteins were incubated with streptavidin agarose beads at 4 °C overnight. The beads were washed extensively for four times using dilution buffer including 0.1% SDS, and the proteins were eluted by boiling with SDS sample buffer for 10 min. The eluted proteins were either resolved in 12% SDS–PAGE for immunoblotting or stained with Commassie to reveal nedd8-modified proteins.

### Enzymatic in-gel digestion

The proteins separated by SDS–PAGE were excised from the gel and the gel pieces containing protein were destained with 50% acetonitrile (ACN) containing 50 mM NH_4_HCO_3_ and vortexed until CBB was completely removed. These gel pieces were then dehydrated in 100% ACN and vacuum-dried for 20 min with SpeedVac. For the digestion, gel pieces were reduced using 10 mM DTT in 50 mM NH_4_HCO_3_ for 45 min at 56 °C, followed by alkylation of cysteines with 55 mM iodoacetamide in 50 mM NH_4_HCO_3_ for 30 min in dark. Finally, each gel pieces were treated with 12.5 ng μl^−1^ sequencing grade modified trypsin (Promega) in 50 mM NH_4_HCO_3_ buffer (pH 7.8) at 37 °C for overnight. Following digestion, tryptic peptides were extracted with 5% formic acid in 50% ACN solution at room temperature for 20 min. The supernatants were collected and dried with SpeedVac. Resuspended samples in 0.1% formic acid were purified and concentrated using C18 ZipTips (Millipore, MA) before MS analysis.

### Liquid Chromatography and Tandem Mass Spectrometry (LC-MS/MS)

The tryptic peptides were loaded onto a fused silica microcapillary column (12 cm × 75 μm) packed with C18 reversed phase resin (5 μm, 200 Å). LC separation was conducted under a linear gradient as follows: a 3–40% solvent B (ACN containing 0.1% formic acid) gradient (solvent A; DW containing 0.1% formic acid), with a flow rate of 250 nl min^−1^, for 60 min. The column was directly connected to LTQ linear ion-trap mass spectrometer (Finnigan, CA) equipped with a nano-electrospray ion source. The electrospray voltage was set at 1.95 kV, and the threshold for switching from MS to MS/MS was 500. The normalized collision energy for MS/MS was 35% of main radio frequency amplitude (RF) and the duration of activation was 30 ms. All spectra were acquired in data-dependent scan mode. Each full MS scan was followed by five MS/MS scan corresponding from the most intense to the fifth intense peaks of full MS scan.

### Database searching and validation

The acquired LC-ESI-MS/MS fragment spectra was searched in the BioWorksBrowser (version Rev. 3.3.1 SP1, Thermo Fisher Scientific Inc., CA) with the SEQUEST search engines against National Center for Biotechnology Information (http://www.ncbi.nlm.nih.gov/) Homo Sapiens database. The searching conditions were trypsin enzyme specificity, a permissible level for two missed cleavages, peptide tolerance; ±2 a.m.u., a mass error of ±1 a.m.u. on fragment ions and variable modifications of carbamidomethylation of cysteine (+57 Da) and oxidation of methionine (+16 Da) residues. The delta CN was 0.1; the Xcorr values were 1.8 (+1 charge state), 2.3 (+2), 3.5 (+3); and the consensus score was 10.15 for the SEQUEST criteria.

### *In vivo* neddylation assay

Identification of neddylated proteins were performed as previously described^35^ with little modification. Briefly, HEK293T cells co-transfected with indicated plasmids for 36–40 h were mock treated or treated with indicated drugs, washed twice with PBS and scraped using 1 ml PBS. About 10% of cell suspension was centrifuged and the cell pellet was lysed in RIPA buffer for western blot analysis. The remaining cell suspension was directly added to 6 ml Guanidinium buffer (6 M guanidinium-HCl, 0.1 M Na2HPO4/NaH2PO4, 0.01 M Tris-Hcl, pH 8.0) containing 5 mM imidazole, 0.1% Triton X-100 and 10 mM β-mercaptoethanol and lysed for 20 min. About 50 μl of Ni-NTA agarose beads were then added directly and the lysates were incubated for 4 h at RT. After incubation, beads were washed once with 800 μl of Guanidinium buffer containing 5 mM imidazole, 0.1% Triton X-100 and 10 mM β-mercaptoethanol, once with 800 μl of Urea buffer A (8 M Urea, 0.1 M Na2HPO4/NaH2PO4, 0.01 M Tris-HCl pH 8.0) containing 5 mM imidazole, 0.1% Triton X-100 and 10 mM β-mercaptoethanol and thrice with 900 μl of Urea buffer B (8 M Urea, 0.1 M Na2HPO4/NaH2PO4, 0.01 M Tris-HCl pH 6.3) containing 5 mM imidazole, 0.1% Triton X-100 and 10 mM β-mercaptoethanol. His-tagged proteins were eluted by incubating the beads in 50 μl elution buffer (5% SDS, 200 mM imidazole, 0.15 M Tris-Cl pH 6.7, 30% glycerol, 0.72 M β-mercaptoethanol, 0.01% Bromophenol Blue) for 20 min. The eluted proteins were directly resolved in SDS–PAGE and blotted to reveal Nedd8 conjugates. For *in vivo* neddylation assay targeting endogenous NEDD8, immunoprecipitation was performed as described in the previous report^48^ with little modification. Briefly, cells transfected with indicated plasmids for 36 h were lysed in denaturing lysis buffer (1% SDS, 20 mM Tris-HCl pH 8.0, 5 mM EDTA, 10 mM iodoacetamide, 250 U ml^−1^ Benzonase (Santa Cruz), protease inhibitors), boiled for 5 min at 90 °C and diluted 10-fold with dilution buffer (20 mM Tris-HCl pH 8.0, 150 mM NaCl, 1% NP-40 and protease inhibitors). Cell lysates were then immunoprecipitated using Flag agarose beads (Sigma) and the precipitates were blotted against anti-NEDD8 antibody.

### Generation of FB–NEDD8 stable cells

U2OS cells transfected with pEF1α-Bir-A were maintained in G418 (neomycin) at 0.5 mg ml^−1^ concentration for several days until cells in parental U2OS cells (U2OS cells without transfection) were completely dead. Medium was changed for every 40 h to remove dead cells, as well as replenish drug sensitivity. Drug-resistant cells were trypsinized, diluted, single clone was selected and the expression level was analysed by immunoblot. The selected U2OS cell line stably expressing Bir-A was transfected with pEF1α-FB–NEDD8 (puromycin-resistant vector) for 40 h before being maintained in a medium containing puromycin at 2 μg ml^−1^ concentration. Non-transfected U2OS cells stably expressing Bir-A were used as control to monitor puromycin drug resistance. A single clone stably expressing FB–NEDD8 similar to endogenous NEDD8 was selected and used for further experiments.

### Generation of sub-stable SRSF3–WT/K85R stable cells

U2OS cells transfected with either flag-tagged pCI-neo-Flag plasmid (empty vector), Flag–SRSF3 WT or Flag–SRSF3–K85R mutant were maintained in G418 (neomycin) at 0.5 mg ml^−1^ concentration along with non-transfected parental U2OS cells. Non-transfected U2OS cells were used as a positive control to check G418 drug sensitivity and expression of Flag-tagged proteins were confirmed and used for further experiments. The stable cell lines were maintained in G418 (at 0.5 mg ml^−1^ concentration) for 48 h at 4-week interval.

### Statistical analysis

All data are means±s.e.m. of at least three independent experiments. Statistical analyses were performed with two-tailed, unpaired Student's *t-*test. *P* value<0.05 was considered statistically significant.

### Data availability

The mass-spectrometry proteomics raw data have been deposited to the ProteomeXchange Consortium via the PRIDE partner repository with the data set identifier PXD004139.

The authors declare that the data supporting the findings of this study are available within the article and its Supplementary Information files.

## Additional information

**How to cite this article:** Jayabalan, A. K. *et al.* NEDDylation promotes stress granule assembly. *Nat. Commun.* 7:12125 doi: 10.1038/ncomms12125 (2016).

## Supplementary Material

Supplementary Information Supplementary Figures 1-18 and Supplementary Tables 1-3

Supplementary Data 1 Proteomic analysis of affinity-purified bands for identifying neddylated proteins
