# Supplementary material for: NEDDylation promotes stress granule assembly
Source: Nat Commun. 2016 Jul 6;7:12125. doi: 10.1038/ncomms12125 (PMC4935812; doi:10.1038/ncomms12125)
Supplement: Supplementary Information — Supplementary Figures 1-18 and Supplementary Tables 1-3 [file ncomms12125-s1.pdf]

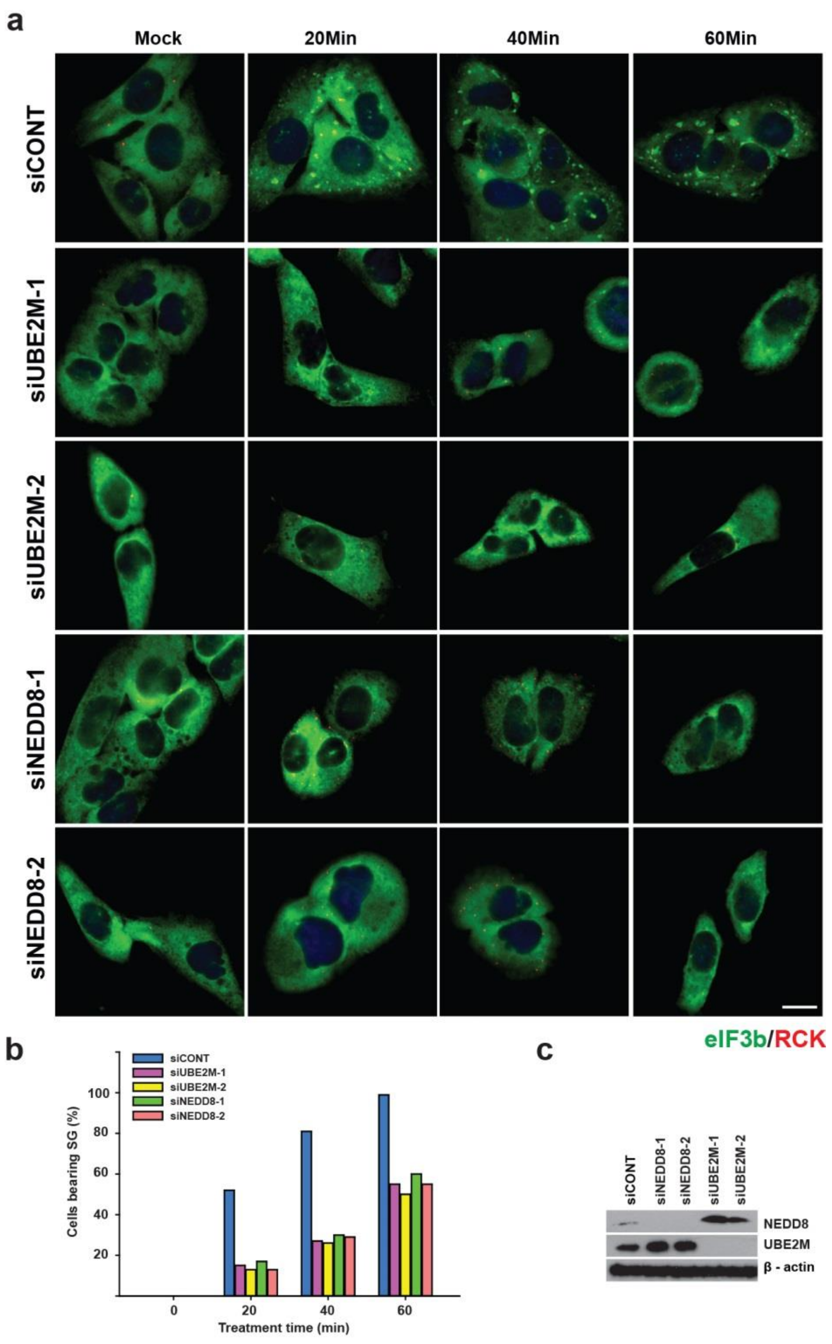

Supplementary Figure 1. SiRNAs targeting NEDD8 and UBE2M inhibit SG assembly. (**a**) siCONT, siUBE2M or siNEDD8 targeting two different sites were transfected prior to treatment with 0.2 mM arsenite in time dependent manner. The presence of SG was visualized by immunostaining against eIF3b (green) and RCK (red). (**b**) Percentage of cells bearing SG is shown as bar graph. (**c**) Immunoblot for knockdown efficiency of NEDD8 and UBE2M. Scale bar, 10 μm.

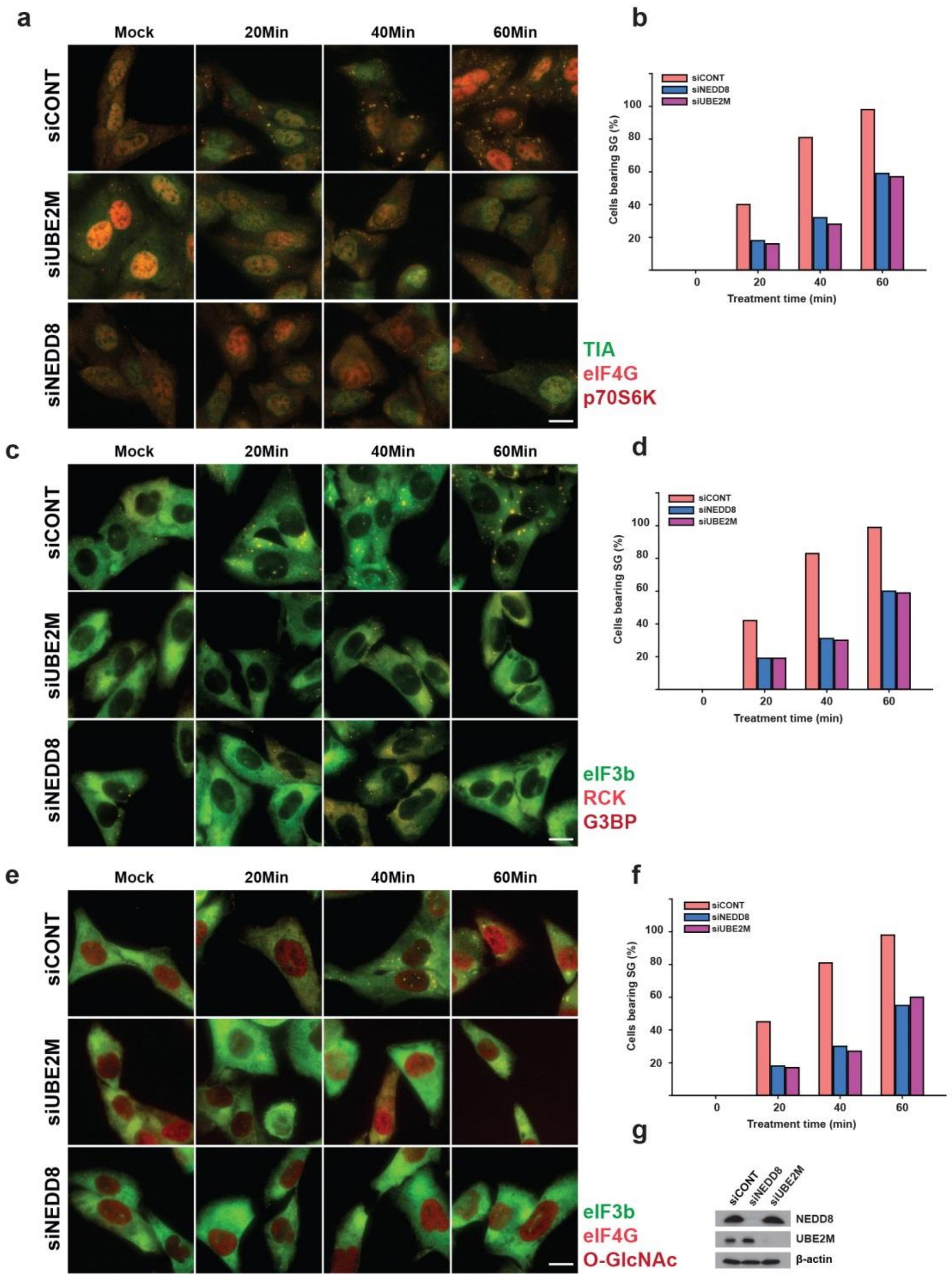

Supplementary Figure 2. Inhibition of SG assembly in NEDD8 and UBE2M depleted cells is tested with different markers. U2OS cells transfected with indicated siRNAs were treated with 0.2 mM arsenite at different time points and presence of SG was evaluated with different established SG markers as follows, (**a**) TIA-1 (green), eIF4G (red), PB marker p70S6K (far red), (**c**) eIF3b (green), SG/PB marker RCK (red), G3BP (far red), and (**e**) eIF3b (green), eIF4G (red), *O*-GlcNAc (far red). (**b,d,f**) Statistical data for percentage of cells bearing SGs. (**g**) Western blot analysis for NEDD8 and UBE2M knockdown efficiency. Scale bar, 10 μm.

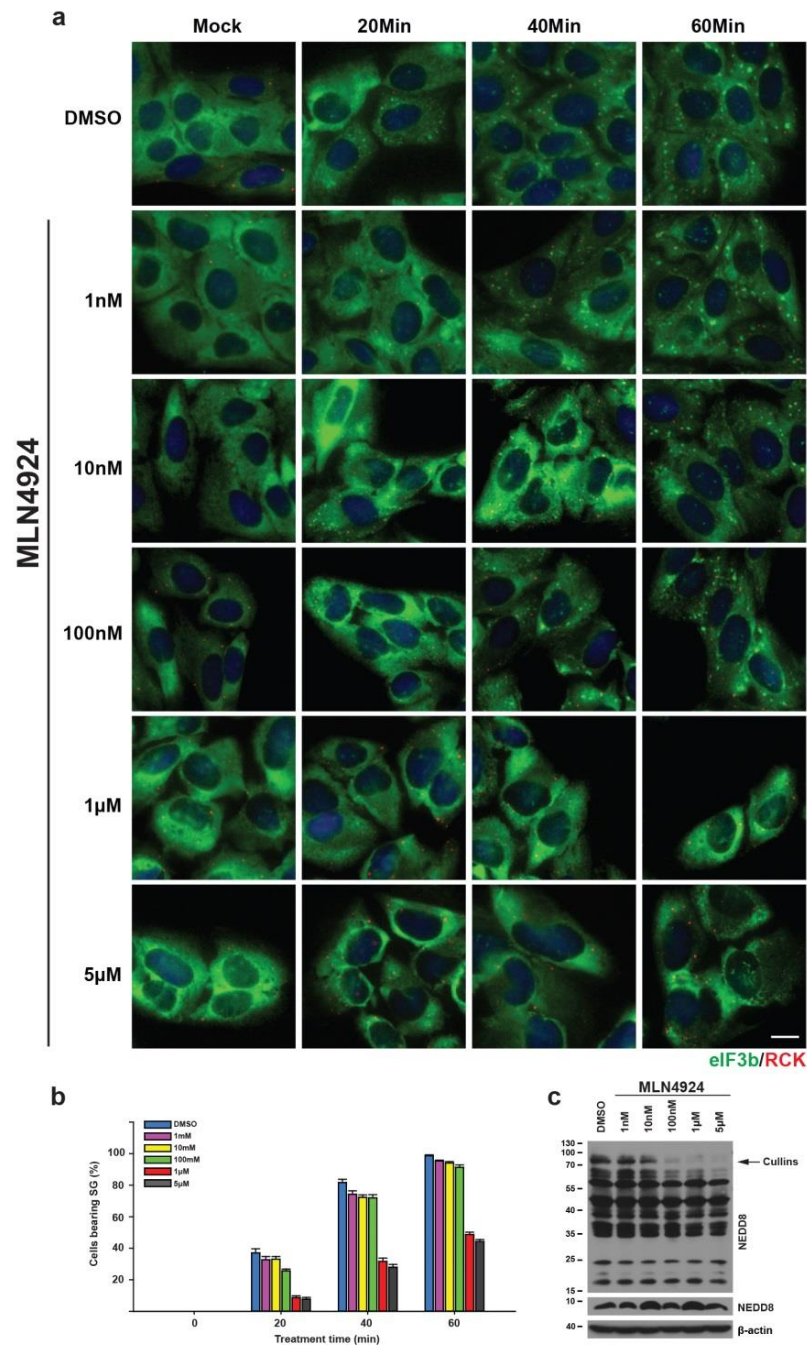

Supplementary Figure 3. MLN4924 pretreatment inhibits SG assembly in dose and time dependent manner. (**a**) U2OS cells grown on coverslips were pre-treated with DMSO or MLN4924 (concentration ranging from 1 nM to 5 μM) for 18 h prior to 0.2 mM arsenite treatment at different time points and then immunostained against eIF3b and RCK. (**b**) Percentage of cells bearing SGs were quantified and represented as bar graph. Error bars indicate s.e.m. (n=3). (**c**) Western blot analysis showing the effect of MLN4924 treatment on total neddylation. Scale bar, 10μm

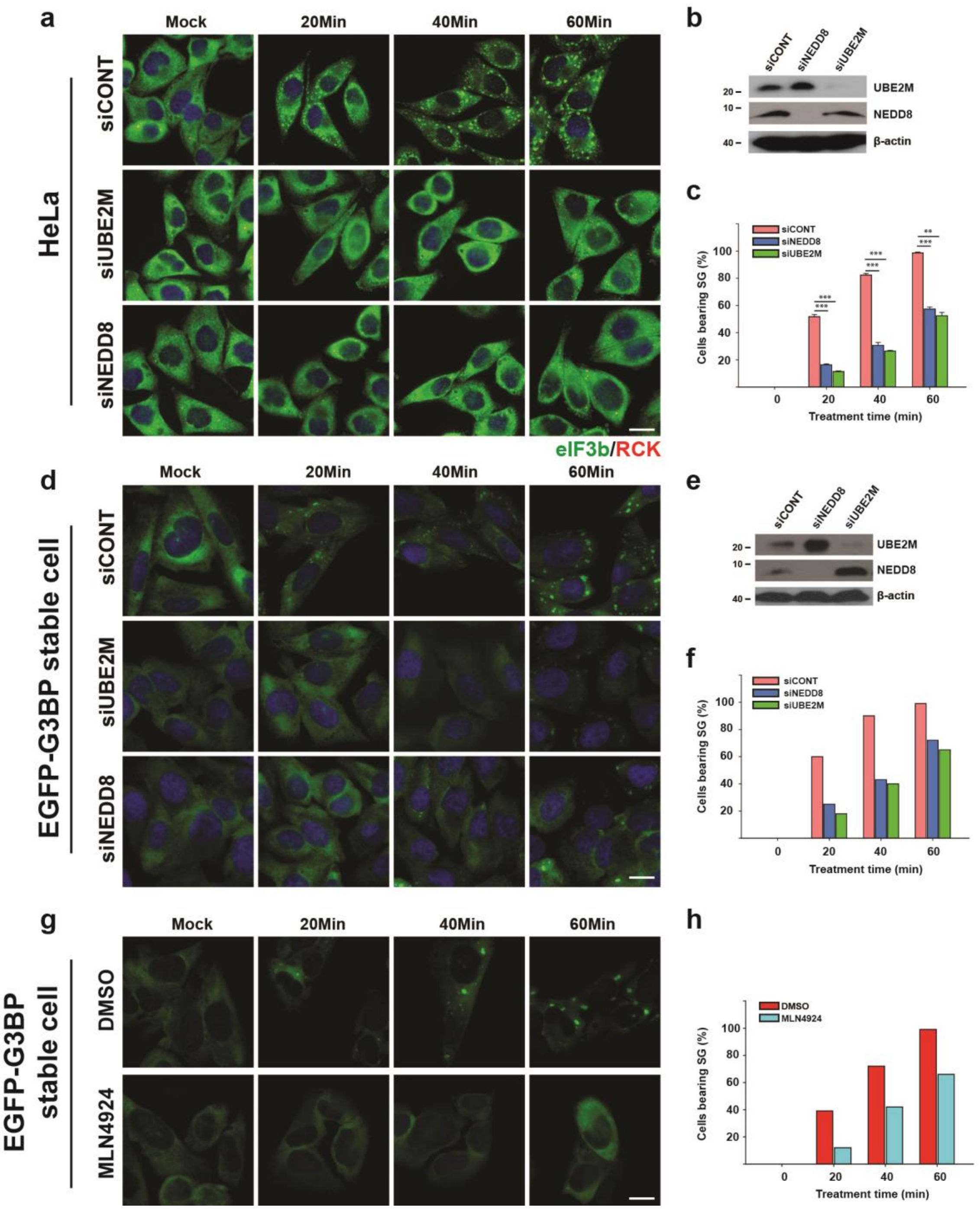

Supplementary Figure 4. Regulation of SG assembly by neddylation pathway occurs in multiple cell lines. (**a**) HeLa cells transfected with indicated siRNAs were treated with 0.2 mM arsenite at different time points and immunostained against eIF3b and RCK. (**b**) Western blot analysis for knockdown efficiency of NEDD8 and UBE2M. (**c**) Percentage of cells bearing SG is shown as bar graph. Error bars indicate s.e.m. (n=3). $^{**}p < 0.01$; $^{***}p < 0.001$, Student's t-test. (**d**) Experiment in (a) was repeated in

stable cell lines expressing EGFP-G3BP. Presence of SG was visualized using GFP immunoflouresence. (**e**) Western blot analysis showing knockdown efficiency of NEDD8 and UBE2M. (**f**) Percentage of cells bearing stress granules is shown as bar graph. (**g**) EGFP-G3BP stable cells were pre-treated with DMSO or MLN4924 (1 μM) for 18 h were treated with 0.2 mM arsenite for indicated time points. (**h**) Statistical data showing percentage of SG inhibition. Scale bar, 10 μm.

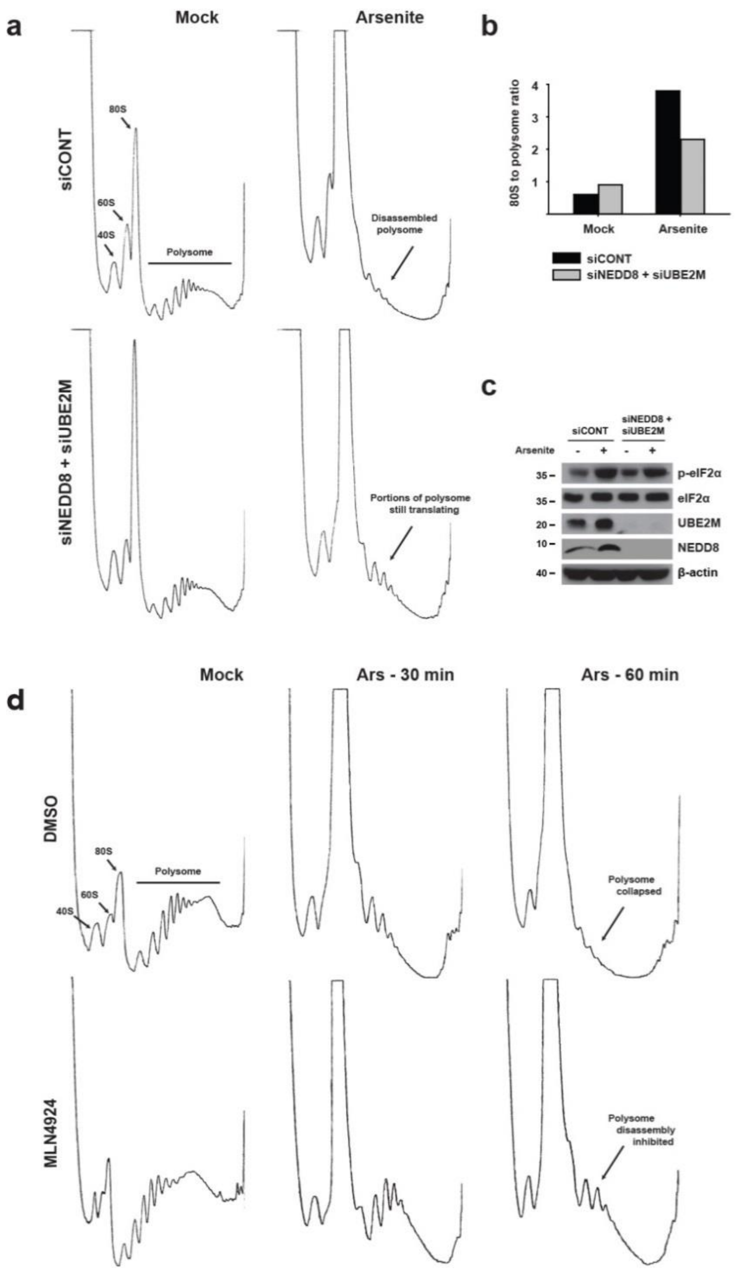

Supplementary Figure 5. Blocking neddylation pathway delays arsenite-induced polysome disassembly. (**a**) U2OS cells transfected with siCONT or mixture of siUBE2M and siNEDD8 were untreated (mock) or treated with 0.2 mM arsenite for 45 mins and subjected to polysome profiling. (**b**) Areas under monosome and polysome curve were quantified and the ratio of 80S to polysome is shown. (**c**) Western blot analysis for knockdown efficiency of NEDD8 and UBE2M. (**d**) U2OS cells pretreated with DMSO or MLN4924 (1 μM) for 18 h were cultured in presence or absence of 0.2 mM arsenite for 30 and 60 mins. Cells were then lysed and subjected to polysome profiling.

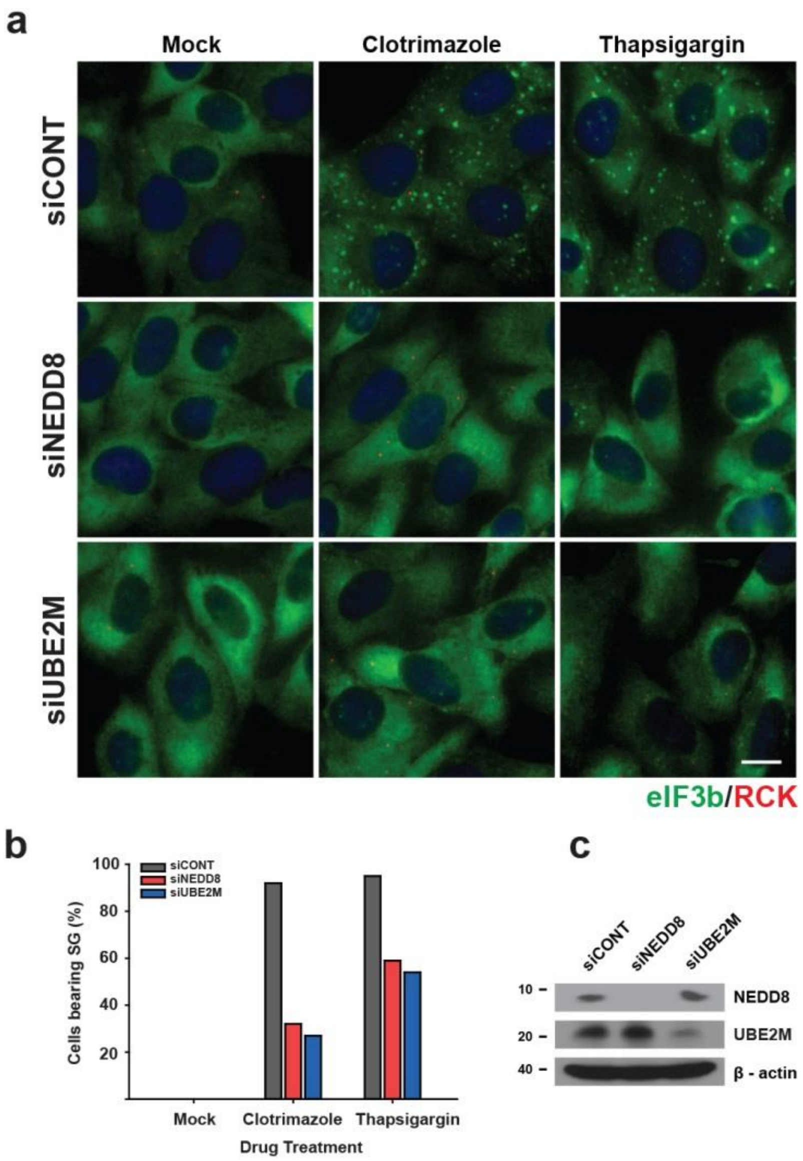

Supplementary Figure 6. Neddylation pathway regulates SG assembly under multiple stress conditions. (**a**) U2OS cells transfected with siCONT, siUBE2M or siNEDD8 were either untreated, treated with clotrimazole (20 µM) or thapsigargin (1 µM) for 45 mins. Cells were then immunostained against eIF3b and RCK antibodies. (**b**) Statistical data showing percentage of cells bearing SG. (**c**) Western data for knockdown efficiency of NEDD8 and UBE2M. Scale bar, 10µm.

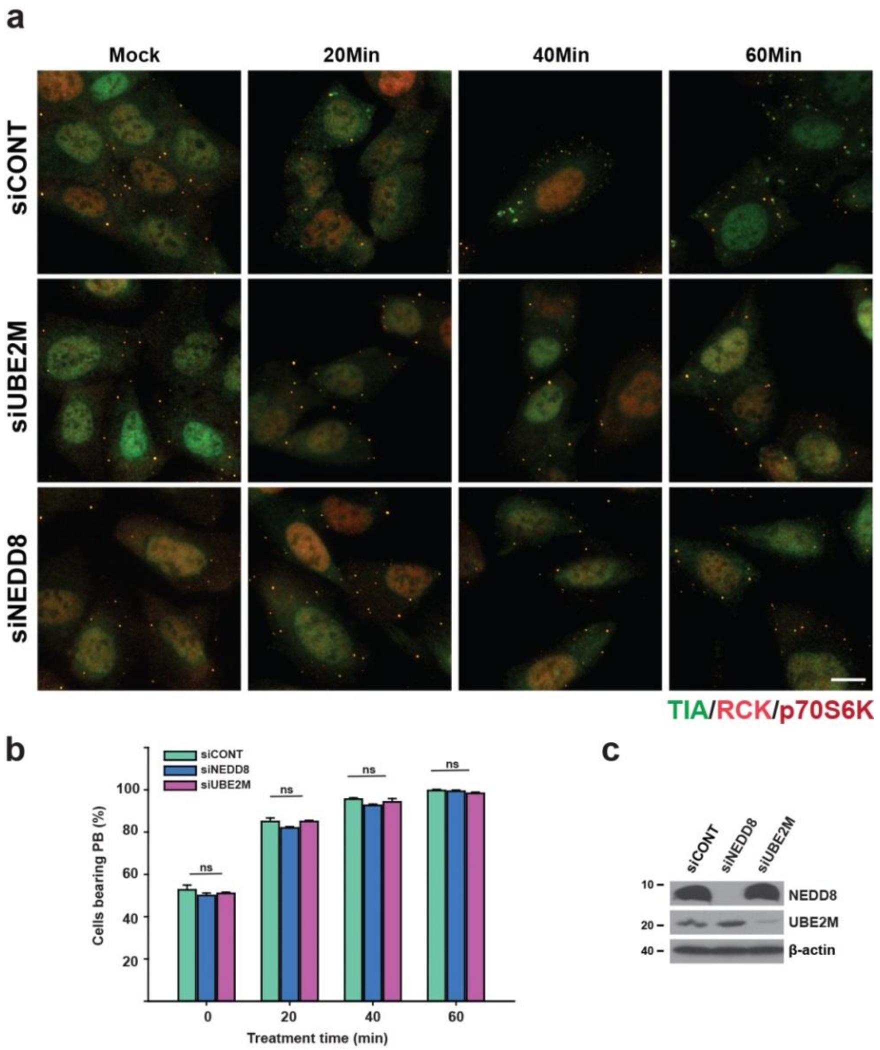

Supplementary Figure 7. Inhibition of neddylation pathway does not affect PB assembly. (**a**) U2OS cells transfected with siCONT, siNEDD8 or siUBE2M were treated with 0.2 mM arsenite at different time points and immunostained against SG marker TIA-1 (green), PB markers RCK (red) and p70S6K (far red). (**b**) Bar graph showing percentage of cells bearing PBs. Error bars indicate s.e.m. (n=3). ns, non-significant, Student's t-test. (**c**) Western blot analysis showing knockdown efficiency of NEDD8 and UBE2M. Scale bar, 10 μm.

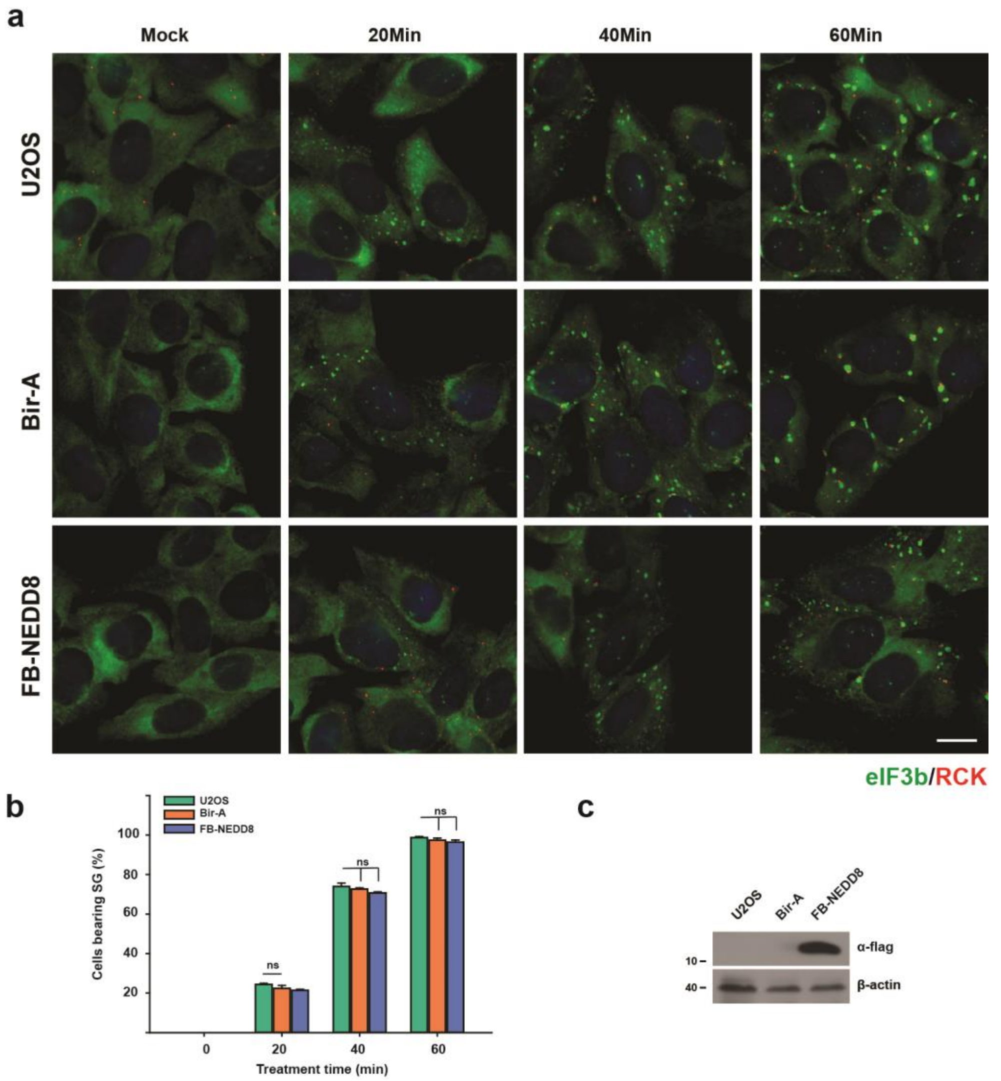

Supplementary Figure 8. Parental U2OS, U2OS cells stably expressing Bir-A and FB-NEDD8 show similar SG assembly pattern. (**a**) Indicated cell lines grown on coverslips were treated with 0.2 mM arsenite at different time points. SG assembly kinetics was analyzed by immunostaining against SG marker eIF3b and PB marker RCK. (**b**) Statistical graph depicting percentage of cells bearing SGs. Error bars indicate s.e.m. (n=4). ns, non-significant, Student’s t-test. (**c**) Western blot analysis showing the expression of FB-NEDD8. Scale bar, 10 μm.

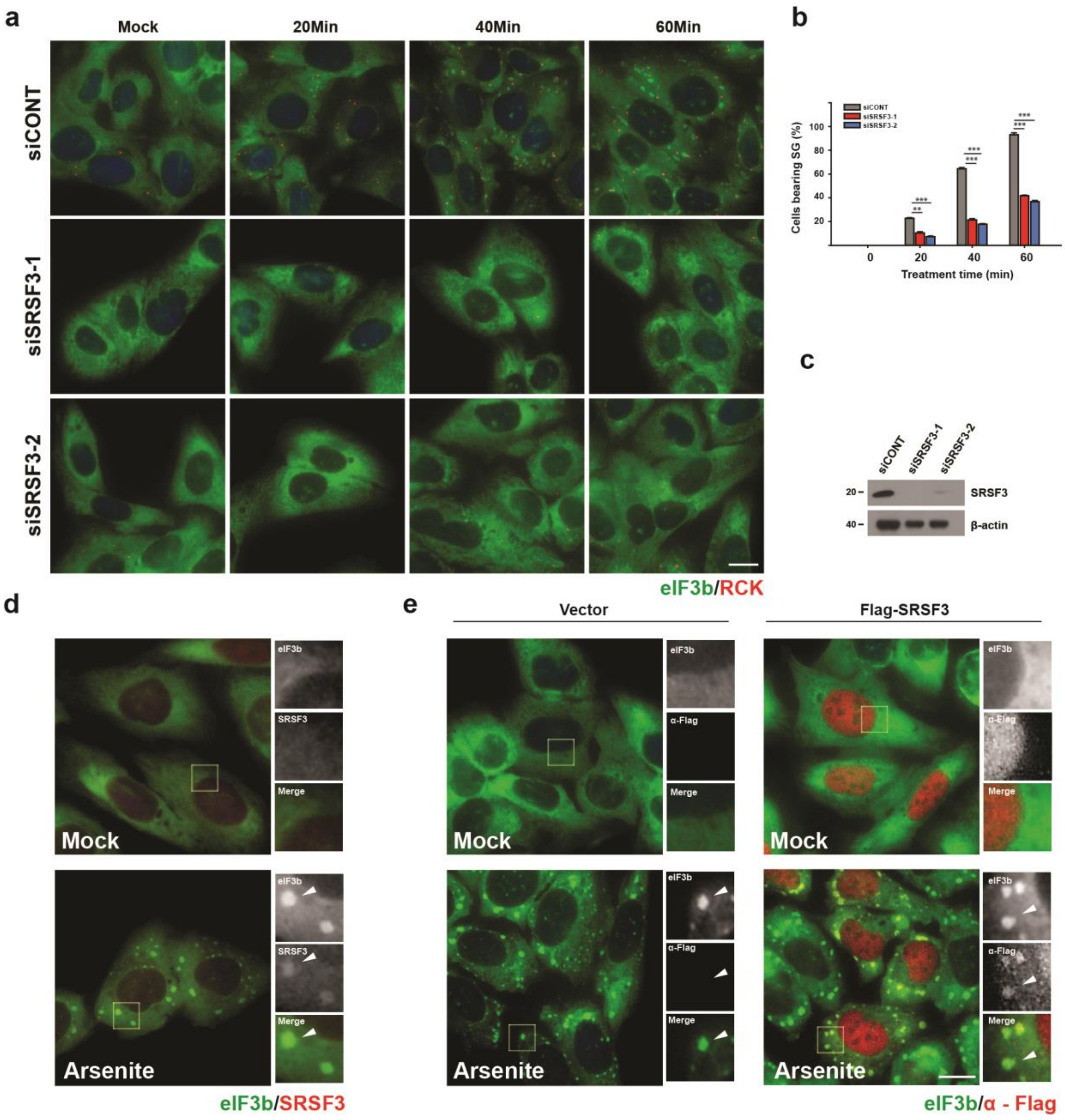

Supplementary Figure 9. SRSF3 is a SG component. (**a**) U2OS cells transfected with siCONT or two siRNAs targeting different sites of SRSF3 were treated with 0.2 mM arsenite and stained against eIF3b and RCK. (**b**) Percentage of cells bearing SGs. Error bars indicate s.e.m. (n=3). $^{**}p < 0.01$; $^{***}p < 0.001$, Student's t-test. (**c**) Immunoblot for SRSF3 knockdown efficiency. (**d**) Endogenous and (**e**) exogenous Flag-SRSF3 is recruited to SGs. U2OS cells either non-transfected or transiently transfected with empty vector or Flag-SRSF3 were treated with 0.5 mM arsenite for 1 h and immunostained against (**d**) eIF3b and SRSF3 or (**e**) eIF3b and Flag antibody. Boxed regions are shown enlarged as both merged and separate colored channel views. White arrow indicates SG. Scale bar, 10 μm.

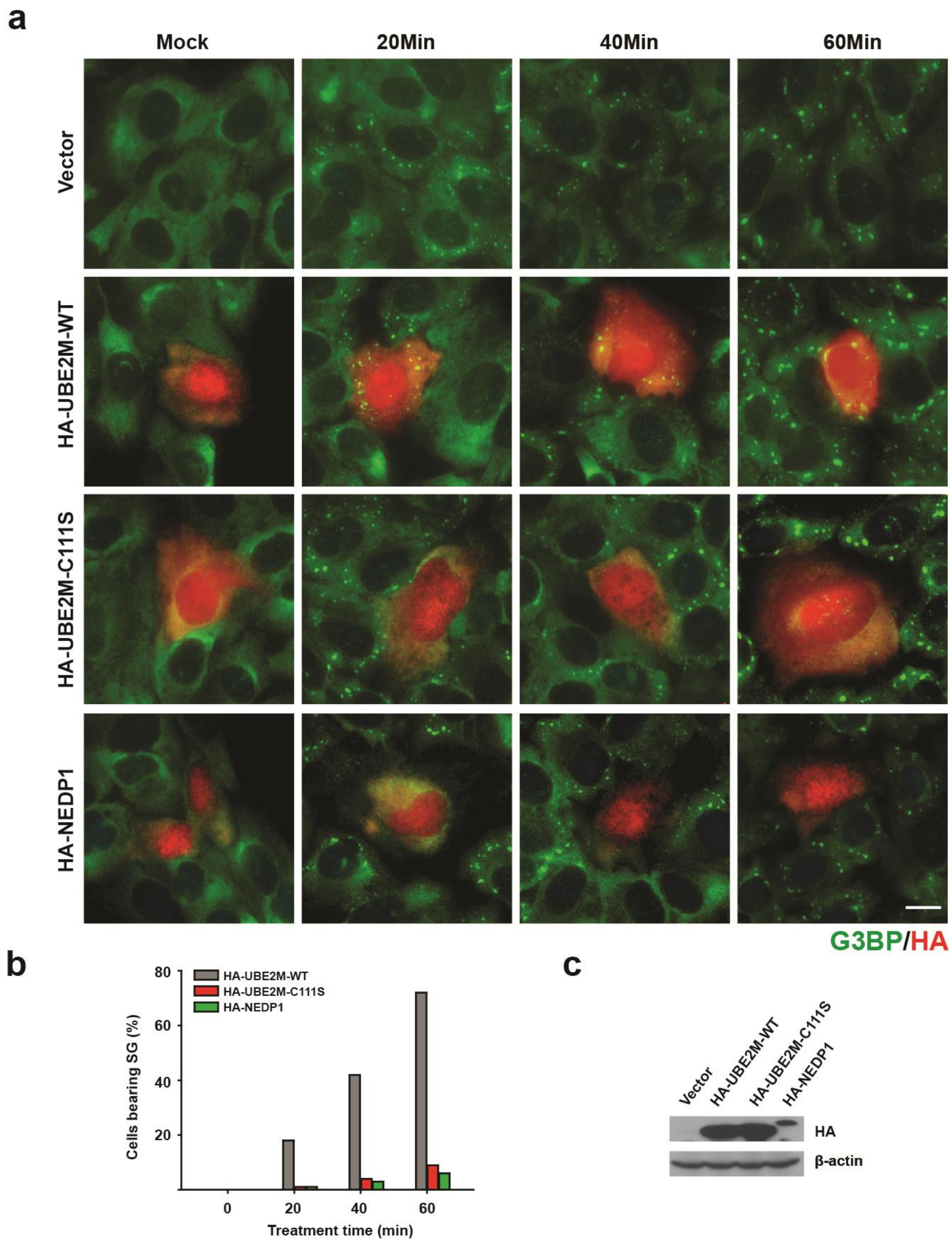

Supplementary Figure 10. Over-expression of catalytically inactive UBE2M (C111S) and NEDP1 inhibit SG assembly. (**a**) U2OS cells transiently transfected with empty vector, HA-tagged UBE2M-WT, UBE2M-C111S or NEDP1 were treated with 0.2 mM arsenite at different time points. Cells were then processed for immunostaining against G3BP and HA. (**b**) Bar graph depicting the percentage of cells bearing SG. (**c**) Western blot analysis showing the expression of transfected constructs. Scale bar, 10 µm.

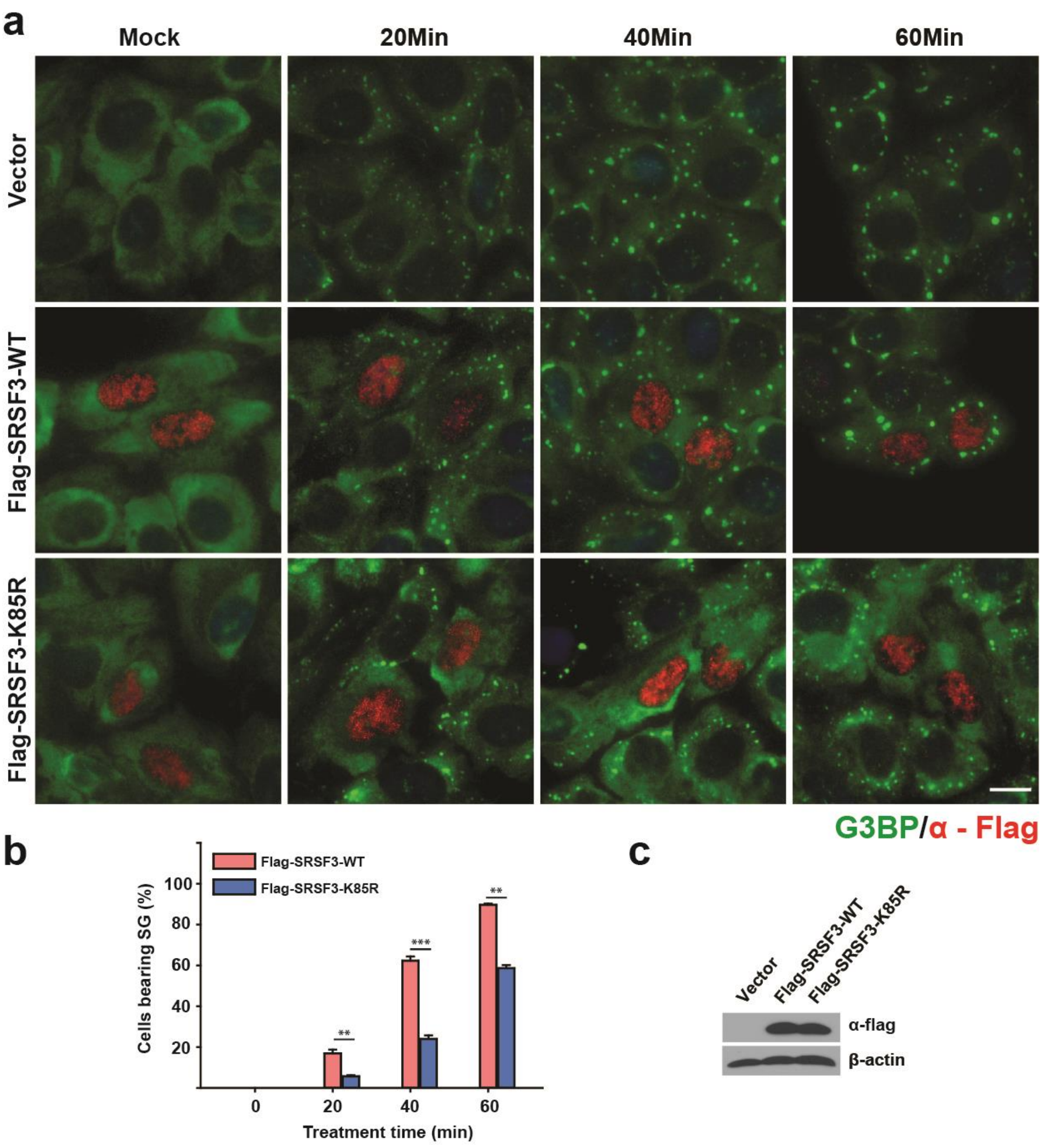

Supplementary Figure 11. Expression of non-neddylatable SRSF3-K85R mutant impairs SG assembly. (**a**) U2OS cells stably expressing empty vector, Flag-SRSF3-WT and Flag-SRSF3-K85R were treated with 0.2 mM arsenite for indicated time points and immunostained against G3BP and Flag antibody. (**b**) Bar graph depicting the percentage of cells bearing SGs in transfected cells. Error bars indicate s.e.m. (n=3). $^{**}p < 0.01$; $^{***}p < 0.001$, Student's t-test. (**c**) Western blot anaysis showing the expression of Flag-tagged SRSF3 constructs. Scale bar, 10 μm.

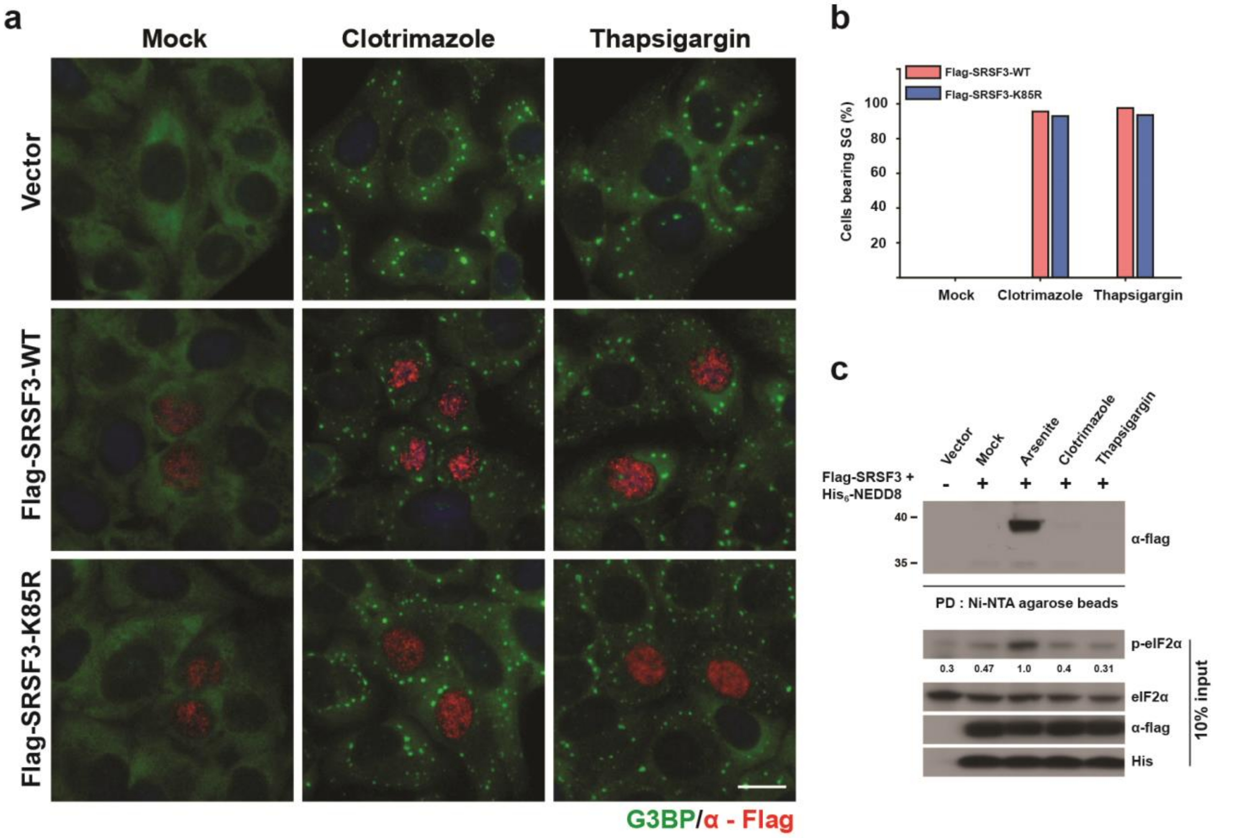

Supplementary Figure 12. Inhibition of SG assembly by SRSF3-K85R and SRSF3 neddylation is arsenite specific. (**a**) U2OS cells transiently transfected with Flag-SRSF3-WT/K85R were treated with 20 μM clotrimazole and 1 μM thapsigargin for 45 mins and stained against G3BP and Flag antibody. (**b**) Percentage of cells bearing SGs in transfected cells. (**c**) HEK293-T cells co-transfected with Flag-SRSF3 and His_6-NEDD8 were treated with 0.5 mM arsenite, 20 μM clotrimazole and 1 μM thapsigargin for 1 h before subjected to neddylation assay. Scale bar, 10 μm.

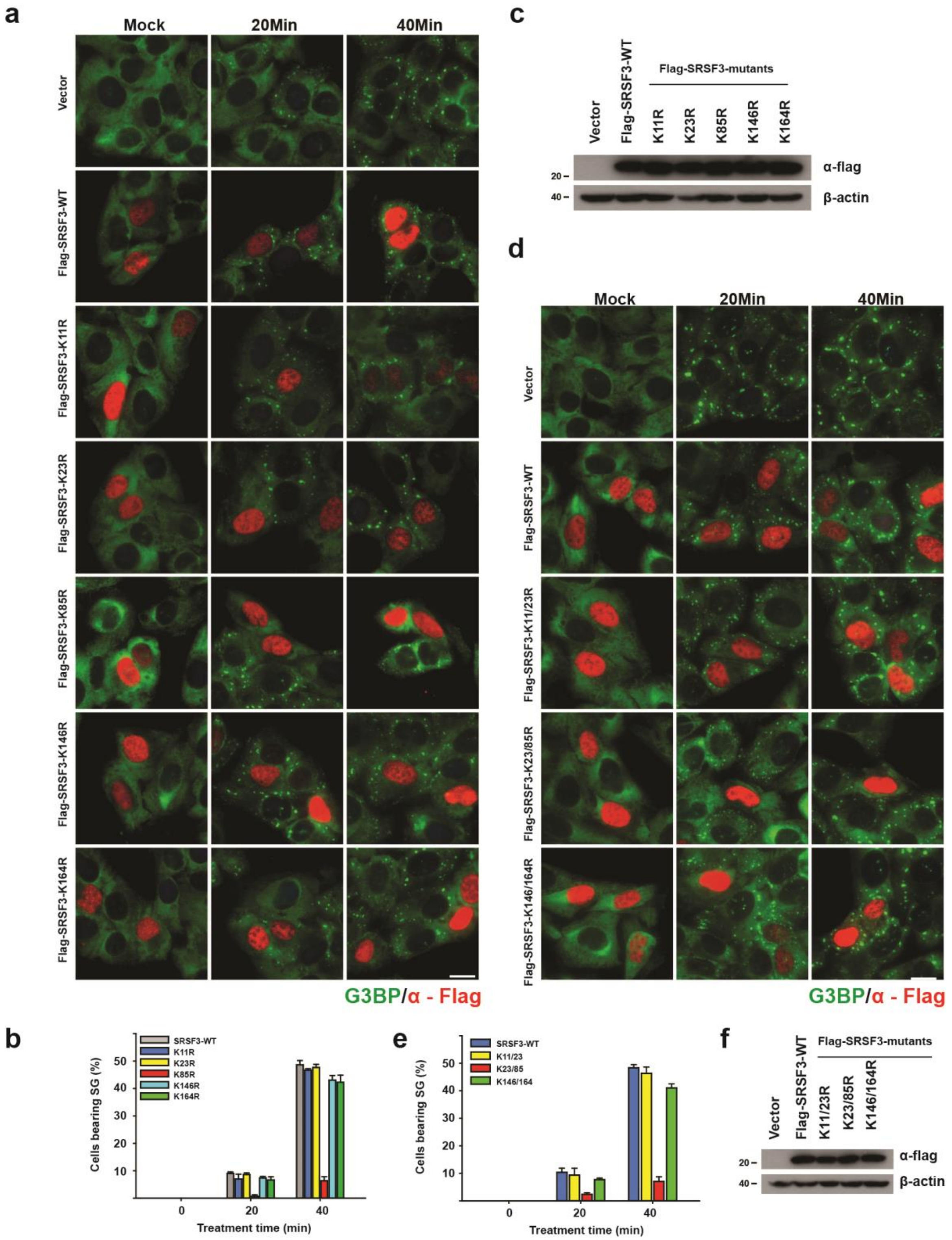

Supplementary Figure 13. SRSF3-K85R but not other lysine mutants inhibits SG assembly. (**a**) U2OS cells transiently transfected with empty vector, Flag-SRSF3-WT or single lysine mutant (11, 23, 85,

146, 164) were treated with 0.2 mM arsenite and stained against G3BP and Flag antibody. (**b**) Bar graph showing the percentage of cells bearing SGs in Flag transfected cells. Error bars indicate s.e.m. (n=3). (**c**) Western blot analysis for the expression of Flag-SRSF3 constructs. (**d**) U2OS cells transiently transfected with empty vector, Flag-SRSF3-WT or double lysine mutant (11/23, 23/85, 146/164) were treated with 0.2 mM arsenite at two different time points before processed for immunostaining. (**e**) Bar graph showing the percentage of cells bearing SGs in transfected cells. Error bars indicate s.e.m. (n=3). (**f**) Western blot analysis for the expression of Flag-SRSF3 constructs. Scale bar, 10µm.

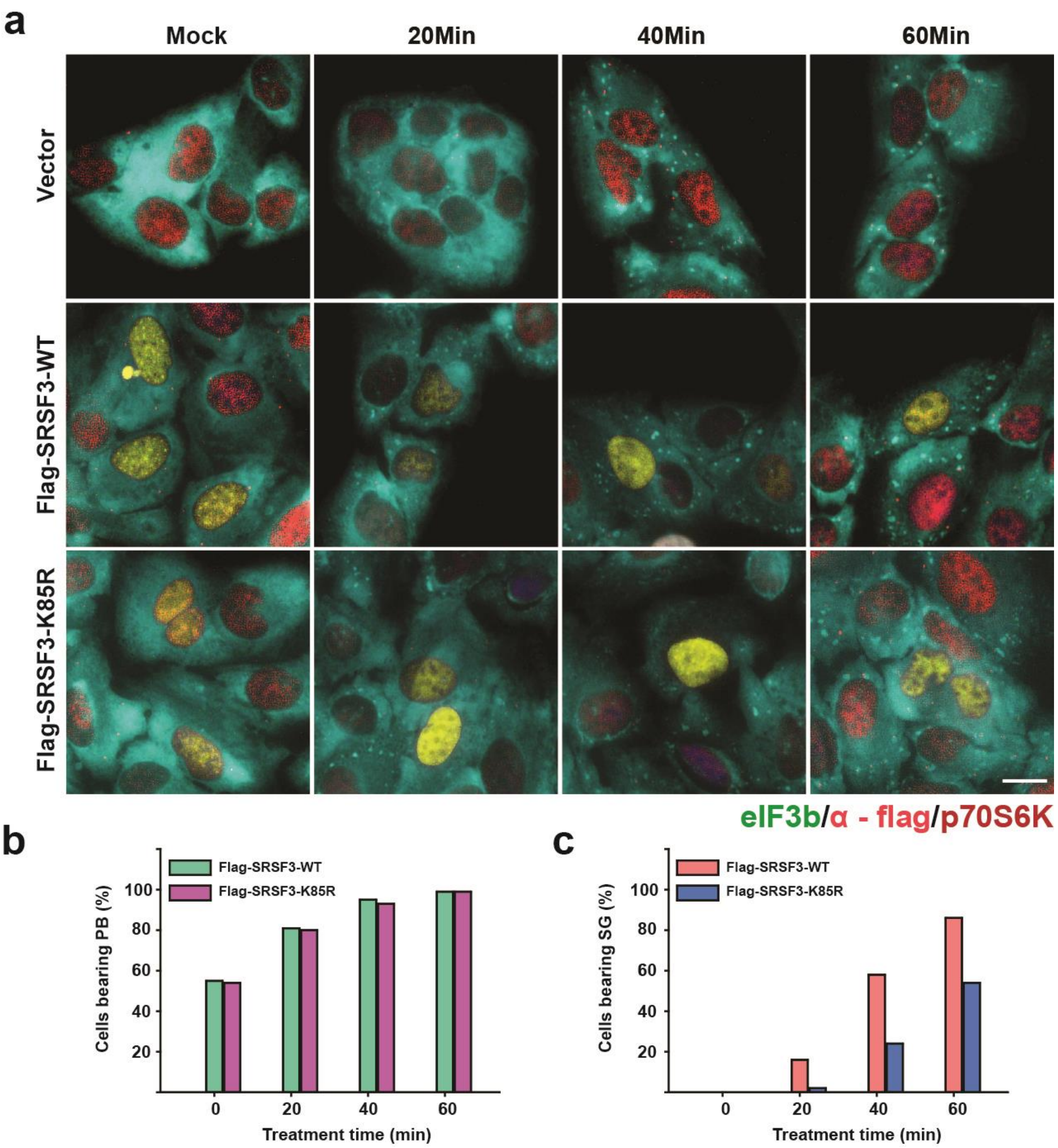

Supplementary Figure 14. SRSF3-K85R mutant does not affect PB assembly. (**a**) Stably expressing empty vector, Flag-SRSF3-WT or Flag-SRSF3-K85R cells were treated with 0.2 mM arsenite and immunostained against eIF3b (green) and p70S6K (red) and Flag antibody (far red). Percentage of cells bearing (**b**) PBs and (**c**) SGs in flag expressed cells were quantified and represented as bar graph. Scale bar, 10 µm.

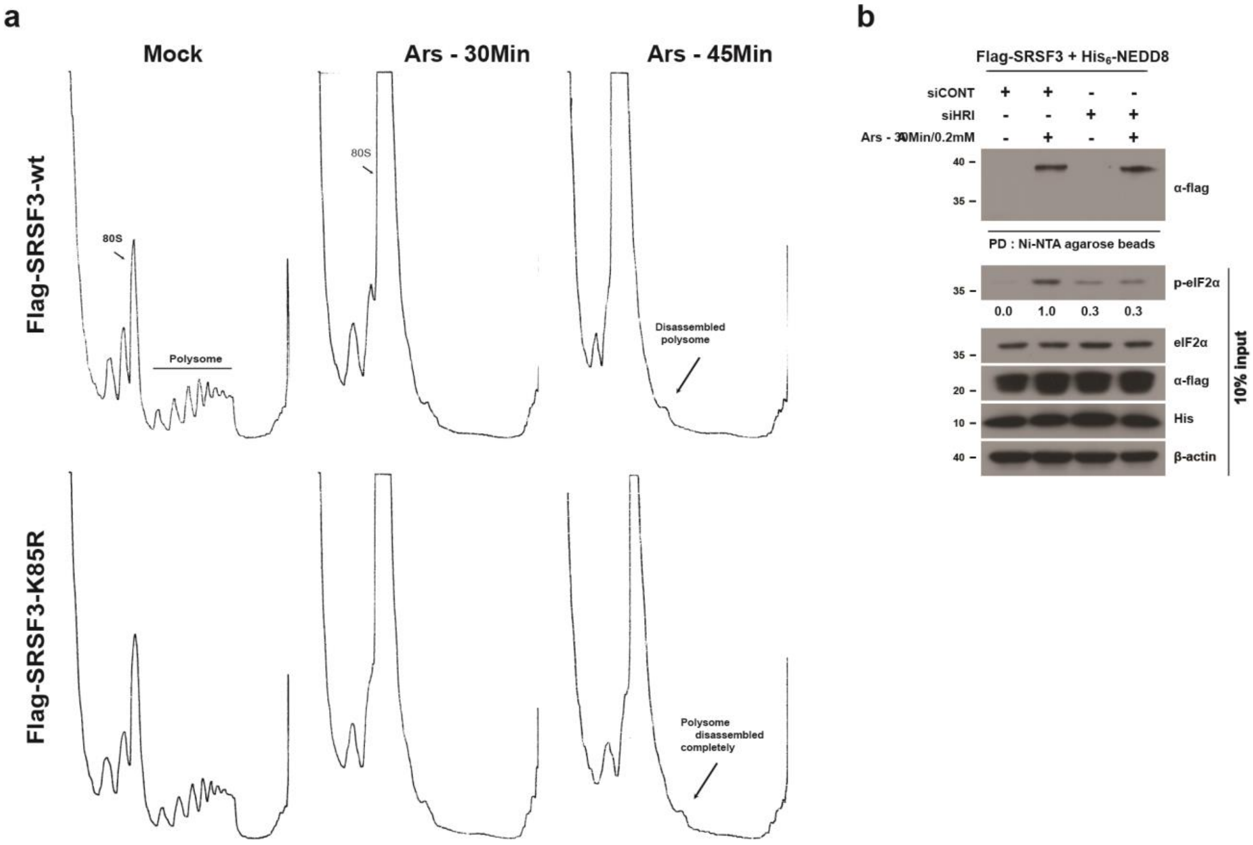

Supplementary Figure 15. SRSF3-K85R mutant does not affect stress-induced polysome disassembly. (**a**) U2OS cells stably expressing Flag-tagged SRSF3-WT and SRSF3-K85R were untreated or treated with 0.2 mM arsenite at two time points (30 and 45mins) and subjected to polysome profiling analysis. (**b**) Knockdown of HRI does not affect stress induced SRSF3 neddylation. siCONT or siHRI transfected cells were subsequently transfected with Flag-SRSF3 and His_6-NEDD8 for 36 hours. Cells were then treated with 0.2 mM arsenite for 30 mins and subjected to *in vivo* neddylation assay.

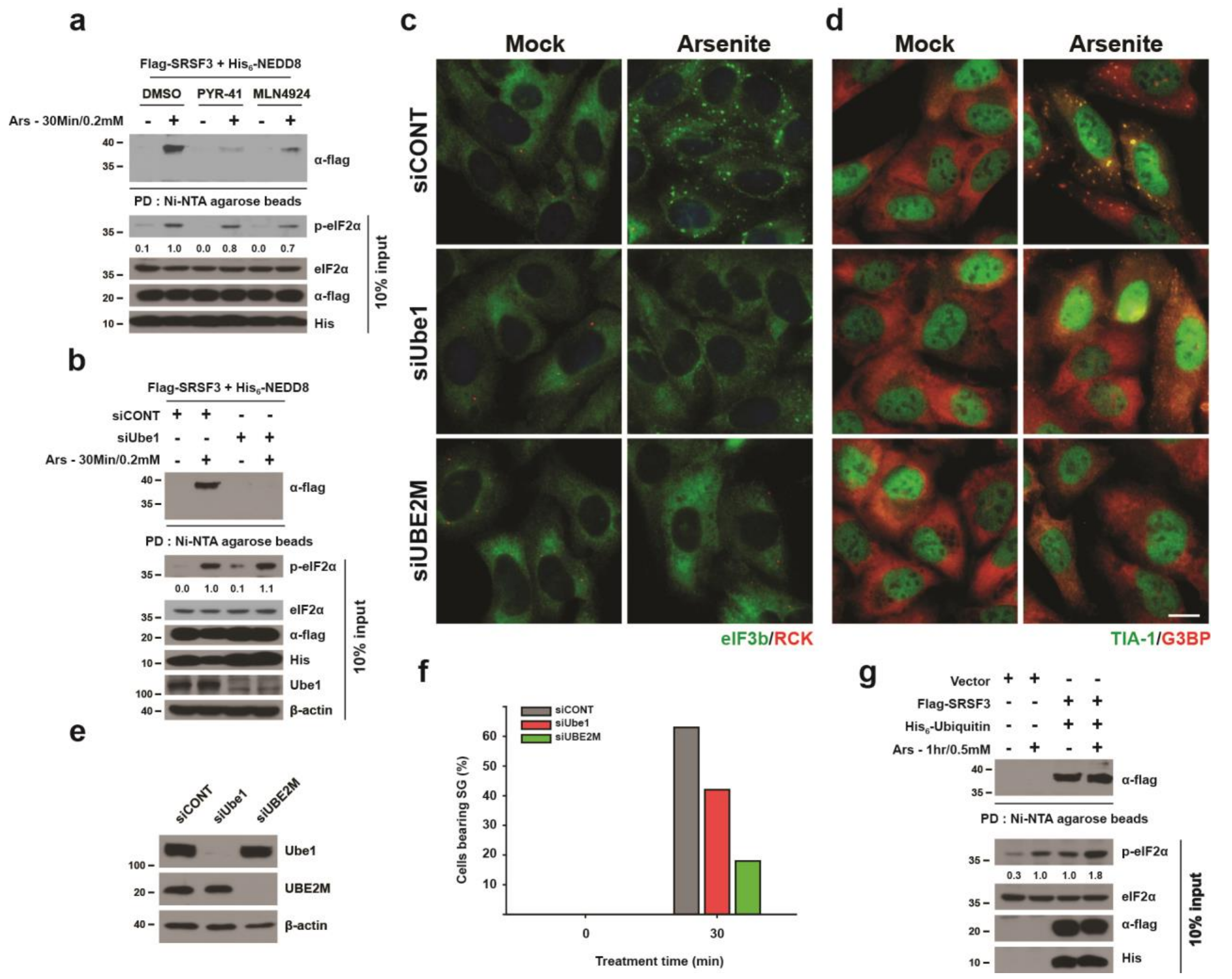

Supplementary Figure 16. PYR-41 and MLN4924 inhibit stress induced SRSF3 neddylation. (**a**) Flag-SRSF3 and His-NEDD8 co-transfected cells were pre-treated with DMSO, 50 nM PYR-41 for 2 h or 1 µM MLN4924 for 18 h. Cells were then treated with 0.2 mM arsenite for 30 mins and subjected to neddylation assay. (**b**) HEK293T cells knocked down with siCONT or siUbe1 were co-transfected with Flag-SRSF3 and His_6-NEDD8. Cells were then treated with 0.2 mM arsenite for 30 mins and *in vivo* neddylation assay was carried out. U2OS cells transfected with siCONT, siUbe1 or siUBE2M were treated with 0.2 mM arsenite for 30 mins and stained against (**c**) eIF3b and RCK, (**d**) TIA-1 and G3BP. (**e**) Western blot analysis for Knockdown efficiency of Ube1 and UBE2M. (**f**) Percentage of cells bearing SGs. (**g**) HEK293T cells transiently transfected with vector or Flag-SRSF3 and His_6-Ubiquitin were mock treated or treated with 0.5 mM arsenite for 1 h and subjected to *in vivo* ubiquitination assay. Scale bar, 10 µm.

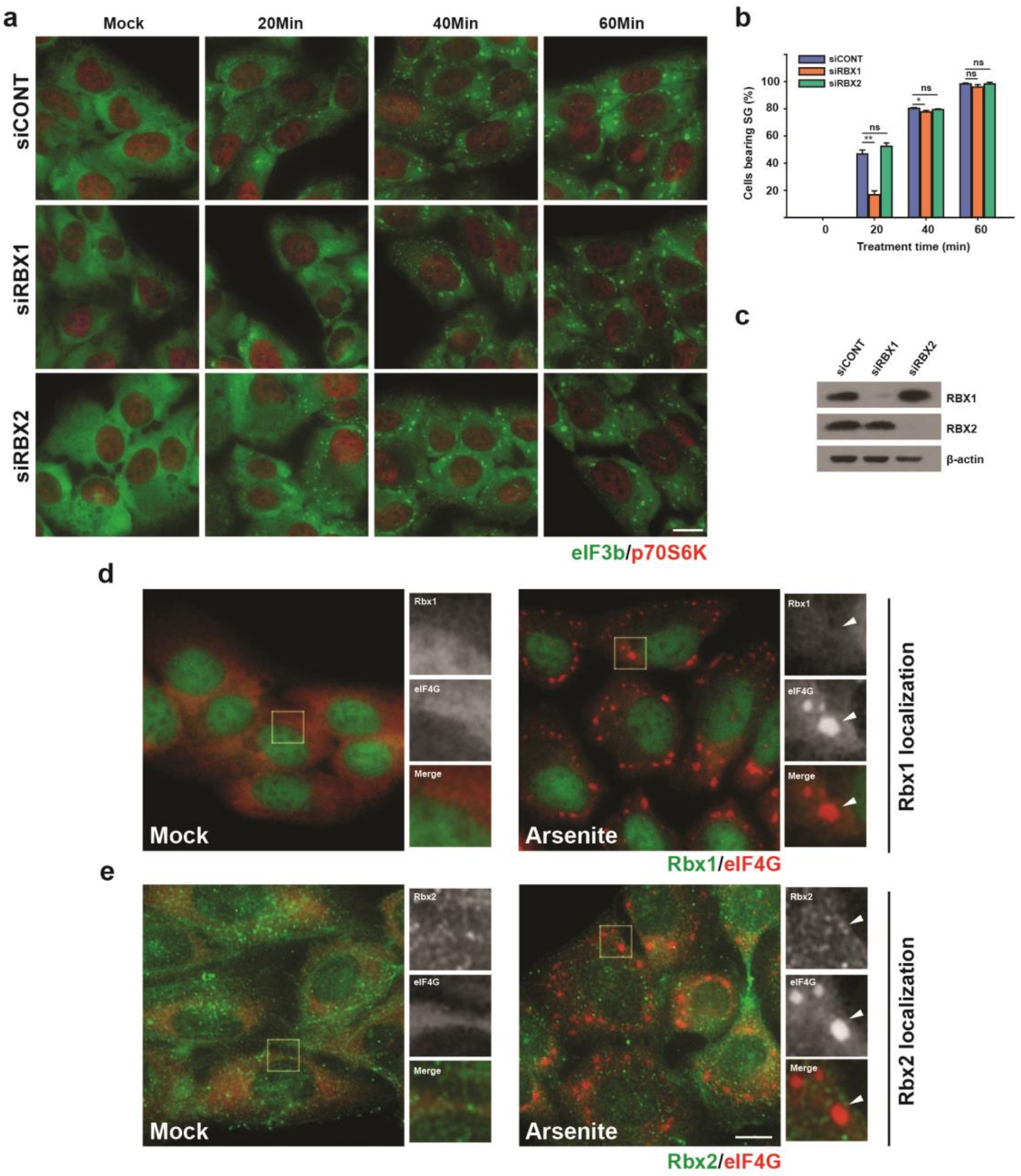

Supplementary Figure 17. RBX1 but not RBX2 knockdown causes minor delay in SG assembly at early stage. (**a**) U2OS cells transfected with siCONT, siRBX1 or siRBX2 were treated with 0.2 mM arsenite for indicated time points and immunostained against eIF3b and p70S6K. (**b**) Statistical graph showing the percentage of cells bearing SGs. Error bars indicate s.e.m. (n=3). $^{*}p < 0.05$; $^{**}p < 0.01$; ns, non-significant, Student's t-test. (**c**) RBX1 and RBX2 knockdown efficiency was confirmed with Western blot analysis. (**d**) RBX1 and (**e**) RBX2 do not localize to SGs. U2OS cells untreated (mock) or treated with 0.5 mM arsenite for 1 hour were immunostained against SG marker eIF4G (green) and RBX1 or RBX2 (red channel) as indicated. Boxed regions are enlarged as both merged and separate colored views. Scale bar, 10 μm.

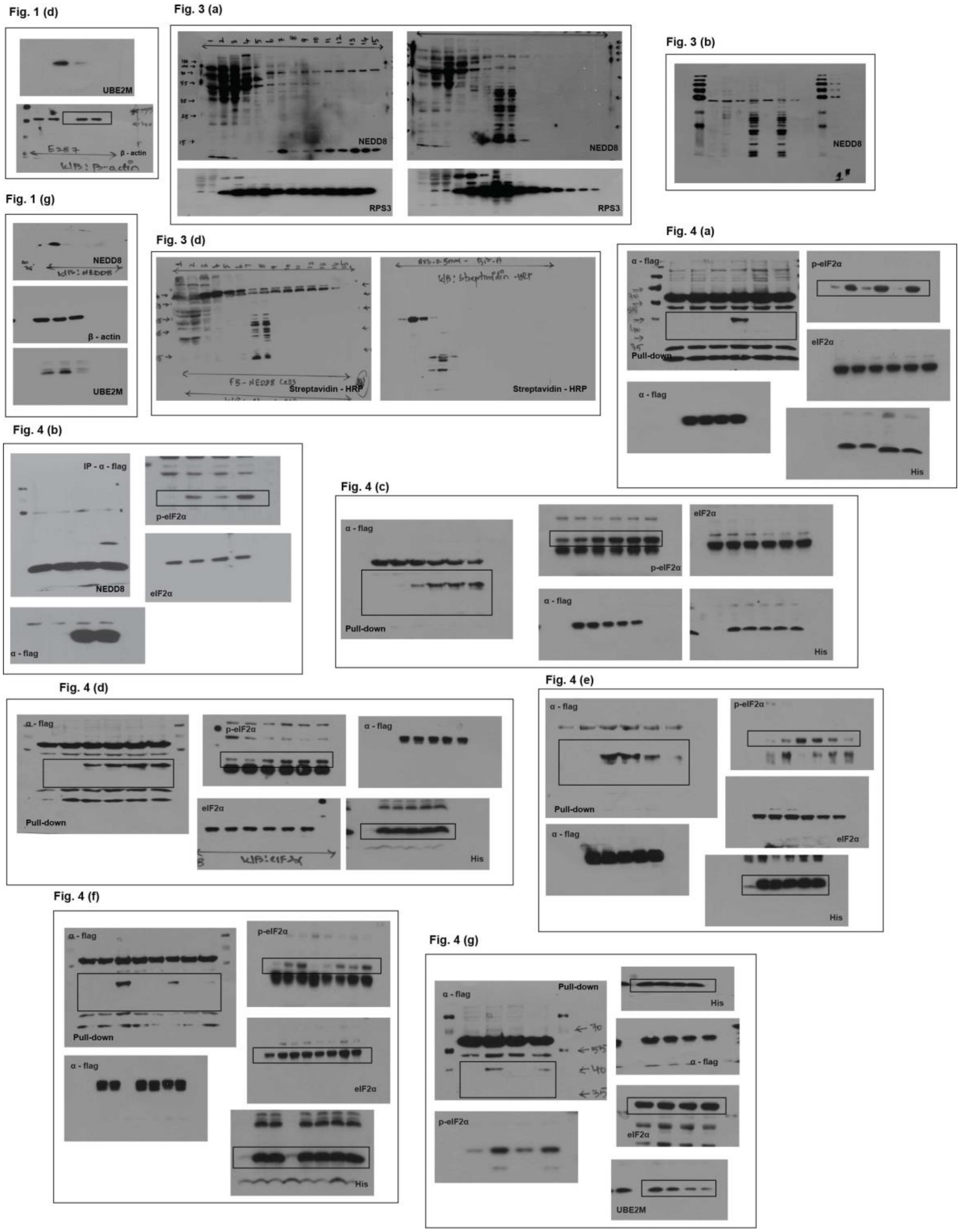

Supplementary Figure 18. Uncropped Western blots

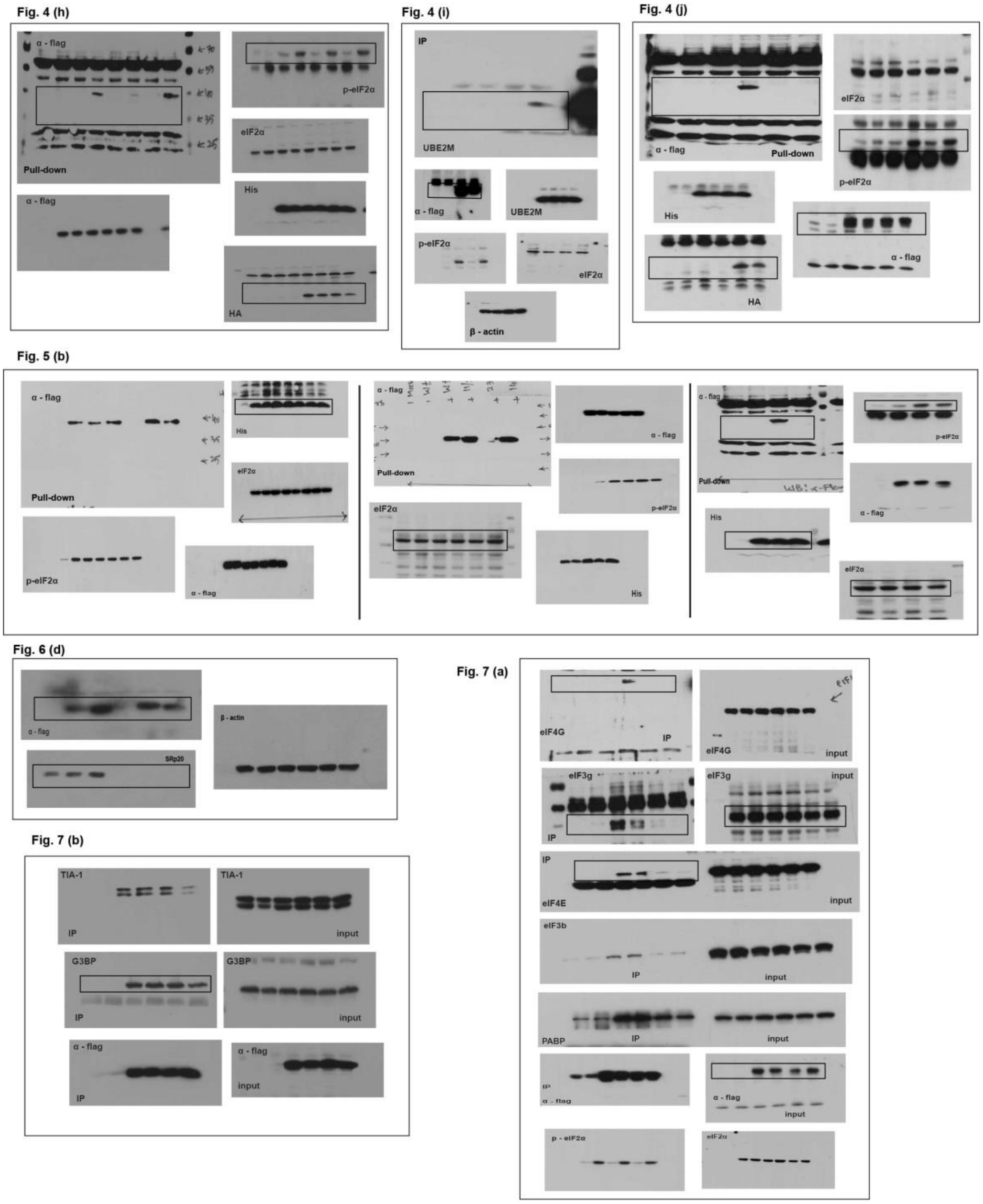

Supplementary Figure 18. Uncropped Western blots (cont.)

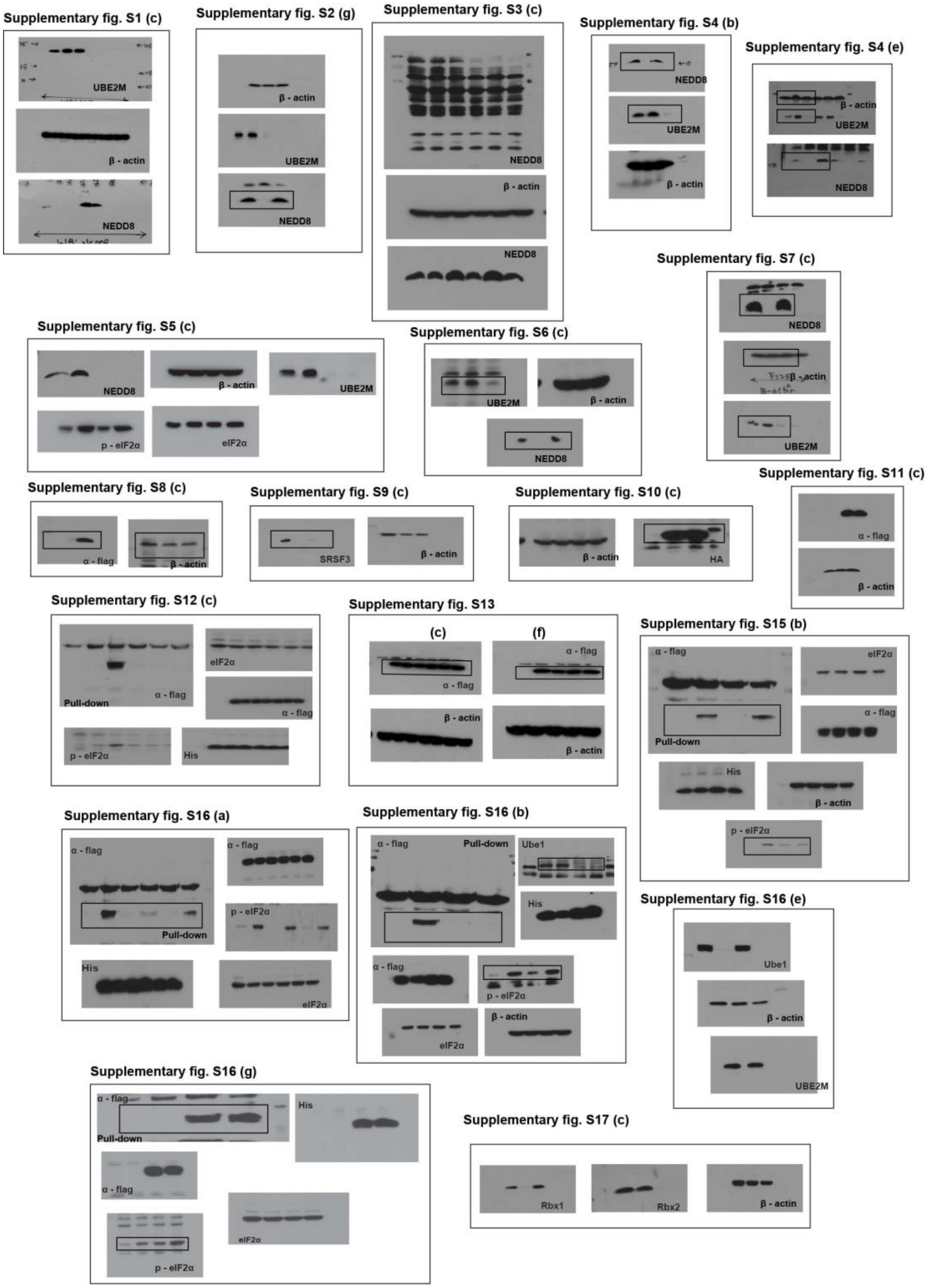

Supplementary Figure 18. Uncropped Western blots (cont.)

Supplementary table – 1: List of siRNA sequences used in this study

siRNA		Target sequence	Source
UBE2M-1		GAAAUAGGGUUGGCGCAUA	Bioneer
UBE2M-2		GAGCUGAACCUGCCCAAGA	Bioneer
NEDD8-1		CAUAAUGAGGCAUCAUAUA	Bioneer
NEDD8-2		GGAGAUUGAGAUUGACAUU	Bioneer
SRSF3-1		GAGUGGAACUGUCGAAUGG	Bioneer
SRSF3-2		AGAGCUAGAUGGAAGAACA	Bioneer
SRSF3-3'UTR		GAAGUGGUGUACAGGAAAU	Bioneer
Rbx-1 (h)	sc-44072	a pool of 3 target specific siRNAs	Santa cruz
Rbx-2 (h)	sc-44073	a pool of 3 target specific siRNAs	Santa cruz
UBE1		GAGAAGCUGGGCAAGCAGAAGUAUU	Bioneer
HRI		GAAGUACACCACCAAUUUA	Bioneer
siCONT		GCAUUCACUUGGAUAGUAA	Bioneer

Supplementary table – 2: List of primer sequences used for cloning in this study

Gene		Vector	Primer sequence
NEDD8	NEDD8_F	Flag-Biotin	CGCGGATCCTCATGCTAATTAAAGTAGAAGAC
	NEDD8_R		CGCGGATCCTCATCCTCCTCTCAGAGCCAA
SRSF3-wt	SRp20_F	Flag	AAAAACGCGTTATGCATCGTGATTCCTGTCCAT
	SRp20_R		AAAAGCGGCCGCCTATTTCCTTTCATTTGACCTA
UBE2M	UBE2M_F	HA	GATCGAATTCATGATCAAGCTGTTCTCG
	UBE2M_R		AATCTCTAGACTATTTCAGGCAGCGCTC
NEDP1	NEDP1_F	HA	TATAGAATTCATGGACCCCGTAGTCTTGAG
	NEDP1_R		GCGC TCTAGA CTA CTT TTT AGC AAG TGT G
SRSF3_Mutants	K11R_F	Flag	CCATTGGACTGTAGGGTTTATGTAGGCAATCTTGG
	K11R_R		CCAAGATTGCCTACATAAACCCTACAGTCCAATGG
	K23R_F		GGAAACAATGGCAACAGGACGGAATTGGAACG
	K23R_R		CGTTCCAATTCCGTCCTGTTGCCATTGTTTCC
	K85R_F		CGAATGGTGAAAGAAGAAGTAGAAATCGTGGC
	K85R_R		GCCACGATTTCTACTTCTTCTTTCACCATTCG
	K146R_F		GGAGAGAAATCACAGGCCGTCCCGATCCTTC
	K146R_R		GAAGGATCGGGACGGCCTGTGATTTCTCTCC
	K164R_F		AAAAACGCGTTATGCATCGTGATTCCTGTCCAT
	K164R_R		ATACGCGGCCGCCTATCTCCTTTCATTTGACC

Supplementary table – 3: List of antibodies used in this study

Antibody against	Dilution		Catalog No.	Species	Source
	WB	ICC			
actin (AC-15)	1:5000		ab6276	ms	abcam
RCK		1:1000	A300-460A	rb	Bethyl
eIF2α (FL-315)	1:1000		sc-11386	rb	santa cruz
p-eIF2α	1:1000		BML-SA405	rb	Enzo
eIF3b (N-20)	1:500	1:200	sc-16377	gt	santa cruz
eIF3g	1:2000		A301-757A	rb	Bethyl
eIF4AII (H-5)	1:1000		sc-137148	ms	santa cruz
eIF4E	1:1000		9742	rb	cell signaling
eIF4G (H-300)	1:1000	1:500	sc-11373	rb	santa cruz
flag	1:1000	1:1000	F3165	ms	Sigma
flag		1:500	F7425	rb	Sigma
G3BP1 (H-10)	1:200	1:500	sc-365338	ms	santa cruz
HA (Y-11)	1:1000	1:200	sc-805	rb	santa cruz
NEDD8	1:1000	1:200	2745	rb	cell signaling
O-GlcNAc		1:1000	MA1-072	ms	Thermo
PABP (F-2)	1:1000		sc-166027	ms	santa cruz
V5	1:1000		R960-25	ms	Invitrogen
His-probe (G-18)	1:500		sc-804	rb	santa cruz
p70S6 Kinase α (H-9)		1:1000	sc-8418	ms	santa cruz
RPS3(D50G7)	1:2000		9538	rb	cell signaling
Rbx1(E-11)	1:1000	1:250	sc-393640	ms	santa cruz
Rbx2(G-8)	1:1000	1:250	sc-166554	ms	santa cruz
SRSF3 (7B4)	1:50	1:100	sc-13510	ms	santa cruz
TIA-1 (C-20)	1:500	1:500	sc-1751	gt	santa cruz
UBE2M (EPR5333)	1:5000	1:500	ab109507	rb	abcam
Ube1	1:2000		A301-126A	rb	Bethyl
Streptavidin-HRP	1:5000		3999		cell signaling
